# ChemPert: mapping between chemical perturbation and transcriptional response for non-cancer cells

**DOI:** 10.1093/nar/gkac862

**Published:** 2022-10-06

**Authors:** Menglin Zheng, Satoshi Okawa, Miren Bravo, Fei Chen, María-Luz Martínez-Chantar, Antonio del Sol

**Affiliations:** Luxembourg Centre for Systems Biomedicine (LCSB), University of Luxembourg, 6 Avenue du Swing, Esch-sur-Alzette, L-4367 Belvaux, Luxembourg; Luxembourg Centre for Systems Biomedicine (LCSB), University of Luxembourg, 6 Avenue du Swing, Esch-sur-Alzette, L-4367 Belvaux, Luxembourg; Liver Disease Laboratory, Center for Cooperative Research in Biosciences (CIC bioGUNE), Basque Research and Technology Alliance (BRTA), Bizkaia Technology Park, Derio, Spain; Centro de Investigación Biomédica en Red de Enfermedades Hepáticas y Digestivas (CIBERehd), 48160 Bizkaia, Spain; German Research Center for Artificial Intelligence (DFKI), 66123 Saarbrücken, Germany; Liver Disease Laboratory, Center for Cooperative Research in Biosciences (CIC bioGUNE), Basque Research and Technology Alliance (BRTA), Bizkaia Technology Park, Derio, Spain; Centro de Investigación Biomédica en Red de Enfermedades Hepáticas y Digestivas (CIBERehd), 48160 Bizkaia, Spain; Luxembourg Centre for Systems Biomedicine (LCSB), University of Luxembourg, 6 Avenue du Swing, Esch-sur-Alzette, L-4367 Belvaux, Luxembourg; DCIC bioGUNE-BRTA (Basque Research and Technology Alliance), Bizkaia Technology Park, 801 Building, 48160 Derio, Spain; IKERBASQUE, Basque Foundation for Science, Bilbao 48013, Spain

## Abstract

Prior knowledge of perturbation data can significantly assist in inferring the relationship between chemical perturbations and their specific transcriptional response. However, current databases mostly contain cancer cell lines, which are unsuitable for the aforementioned inference in non-cancer cells, such as cells related to non-cancer disease, immunology and aging. Here, we present ChemPert (https://chempert.uni.lu/), a database consisting of 82 270 transcriptional signatures in response to 2566 unique perturbagens (drugs, small molecules and protein ligands) across 167 non-cancer cell types, as well as the protein targets of 57 818 perturbagens. In addition, we develop a computational tool that leverages the non-cancer cell datasets, which enables more accurate predictions of perturbation responses and drugs in non-cancer cells compared to those based onto cancer databases. In particular, ChemPert correctly predicted drug effects for treating hepatitis and novel drugs for osteoarthritis. The ChemPert web interface is user-friendly and allows easy access of the entire datasets and the computational tool, providing valuable resources for both experimental researchers who wish to find datasets relevant to their research and computational researchers who need comprehensive non-cancer perturbation transcriptomics datasets for developing novel algorithms. Overall, ChemPert will facilitate future *in silico* compound screening for non-cancer cells.

## INTRODUCTION

The inference of the relationship between chemical perturbations and their specific transcriptional response has wide biological and clinical relevance, such as drug discovery. However, the inference of such relationship using computational models of signal transduction remains a challenge, as they require data for different molecular regulatory layers, such as phospho-proteomics data, which are not widely available. On the other hand, the analysis of transcriptomics changes before and after perturbations enables us to directly map the chemical perturbations to their response genes. However, a major limitation is that such transcriptional changes (i.e. transcriptional signatures) are usually cell specific and need to be generated for each cell type of interest, necessitating a large compendium of gene expression profiles for large-scale drug screening.

In an effort to address this important challenge, the Connectivity Map (CMap) project and more recently, the LINCS L1000 project, have collected gene expression profiles for thousands of perturbagens at different time points and doses in different cell lines (1,2). These resources have been successfully employed for various studies (3,4). In addition, they offer computational tools for drug prediction based on GSEA of query genes. A similar approach has been proposed for identifying chemical compounds for enhancing cellular reprogramming (5). However, the majority of the gene expression profiles in these compendia consist of cancer cell lines, which are known to exhibit signal transduction pathways and gene regulatory networks that are significantly different from those of non-cancer cells (6). For this reason, we hypothesize that the gene expression profiles in these resources are not optimal for addressing the challenges related to transcriptional responses in non-cancer cells, such as those in non-cancer disease, immunology and aging.

In this study, we present ChemPert (https://chempert.uni.lu/), the first comprehensive compendium of manually curated transcriptional signatures derived solely from non-cancer cell perturbation datasets, combined with a tool that allows users to predict either the transcriptional responses of perturbations or chemical compounds targeting desired sets of transcription factors (TFs). The chemical perturbations in ChemPert are denoted as perturbagens, which include both chemical and biological agents such as small molecules, drugs, cytokines and growth factors. ChemPert consists of 82 270 transcriptional signatures of 167 unique non-cancer cell types perturbed with 2566 unique perturbagens. Unlike the existing approaches that predict chemical compounds directly from a database (1,2), ChemPert first predicts signalling proteins and then identifies potential perturbagens targeting these proteins. This approach allows for the identification of novel perturbagens that are not contained in the collected transcriptional compendium.

We show that predictions generated for non-cancer cells when using ChemPert database were significantly more accurate than those based on cancer databases, underscoring the importance of non-cancer cell perturbation datasets collected in this study. Our benchmarking also reveals that considering initial cell states in addition to perturbagen similarity for TF response prediction results in significantly higher predictive accuracy than using perturbagen similarity alone. To further demonstrate the practical utility of ChemPert, we applied it to the RNA-seq data of non-alcoholic steatohepatitis (NASH) models, which predicted the differential TF responses to chemical drugs for NASH and these predicted response TFs were in agreement with the functional effects of the drugs on different stages of NASH. In another application, ChemPert was able to predict potential novel pharmacologic therapeutics for osteoarthritis (OA). Notably, no effective pharmacologic treatments are currently available for OA and the predicted perturbagens constitute potential novel therapeutics that could be further experimentally validated.

The ChemPert web interface is user-friendly and allows easy access and download of the entire datasets. The computational tool is also embedded in ChemPert as a webtool and can easily be run by users through the web interface. ChemPert will serve as valuable resources for not only experimental researchers who wish to find previous datasets relevant to their research, but also computational researchers who aim to develop new algorithms that require a large amount of non-cancer perturbation transcriptomics data. Overall, ChemPert provides a comprehensive non-cancer cell perturbation compendium and facilitates future *in silico* predictions of perturbation response and chemical compound discovery for inducing desired effects on non-cancer cells.

## MATERIALS AND METHODS

### Construction of ChemPert database

**Figure 1.**
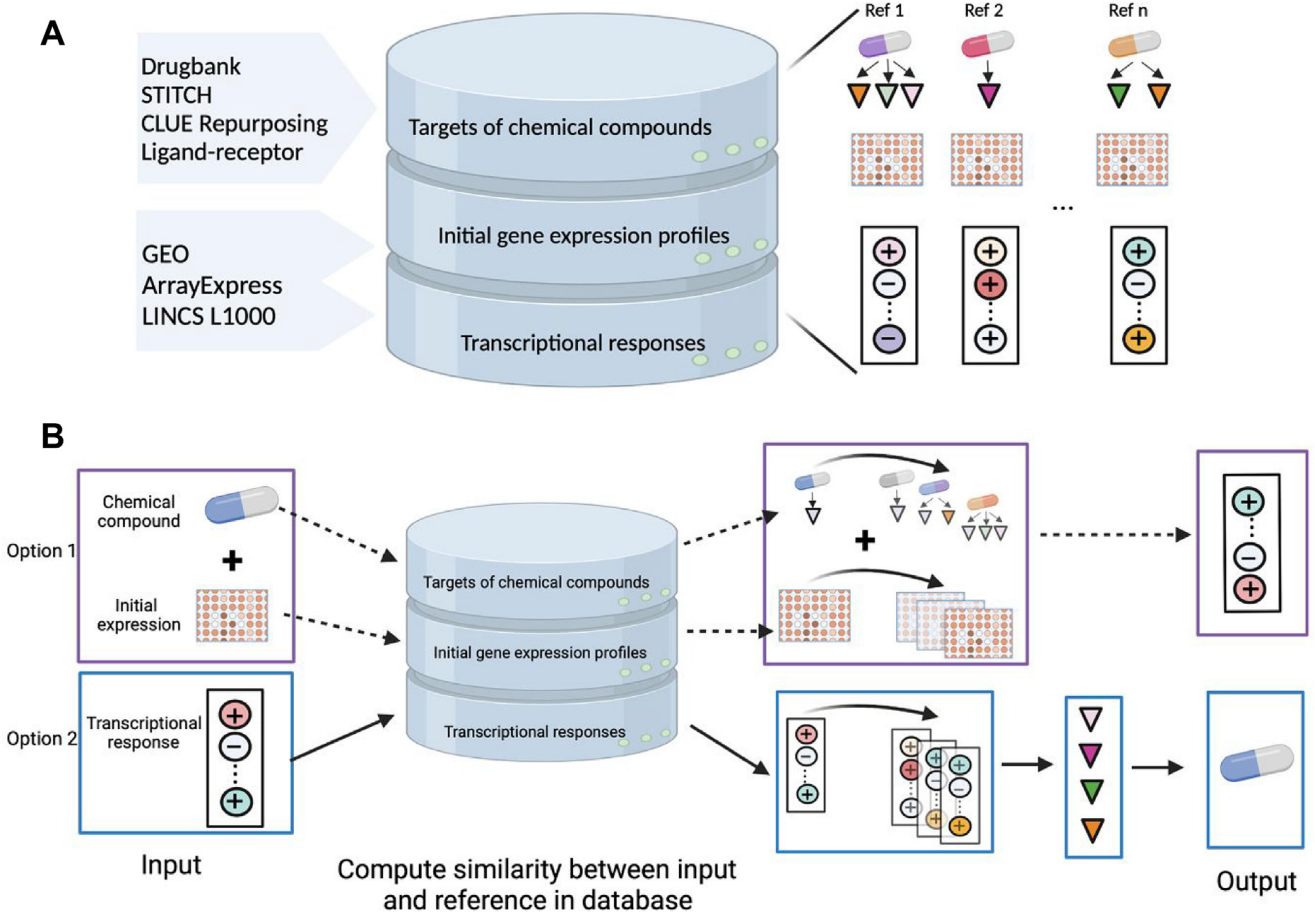
Schematic outline of ChemPert. (**A**) The sources and three main components of ChemPert database. (**B**) Illustration of built-in algorithms in ChemPert. One option for predicting the TF responses given the perturbagen and expression profile of initial cellular state (Option 1) and the other for predicting perturbagens that induce desired transcriptional response (Option 2).

In this study, we constructed a database depicting the relationship between chemical perturbations, protein targets of perturbations and downstream transcriptional signatures. We considered the responses of transcriptional regulators including transcription factors, transcriptional co-factors and chromatin remodelling factors as ‘response TFs’ to refer to these gene products for brevity. First, we collected transcriptome profiles of chemical perturbations (including small molecules, growth factors, cytokines and other protein ligands) from Gene Expression Omnibus (GEO) (7) and ArrayExpress (8). Specifically, the keywords commonly used in perturbation studies, such as ‘time series’, ‘response’, ‘treat’, ‘perturb’, ‘presence’ and ‘effect’, were used to search for the datasets in GEO and ArrayExpress. Then, we manually curated the datasets focusing on non-cancer cell types/lines or tissues in human, mouse and rat (Figure 1A). The datasets were pre- processed, including background correction and normalization, either with the same approaches from the original studies or using the limma R package (v3.38.3) (9). In addition, we also extracted the chemical perturbation datasets of non-cancer cells from LINCS L1000 at Level 3, where the quantile normalization was performed (2). The response TFs of each perturbagen were obtained by performing differential expression analysis using the limma R package. The genes with Benjamini-Hochberg (BH) adjusted *P*-value ≤0.05 and absolute fold change ≥1.5 were considered as differentially expressed genes (DEGs) compared to unperturbed control samples when the sample replicates were larger than two. Otherwise, only the fold change was used as the criterion. Differentially expressed TFs were considered as response TFs based on the annotations from AnimalTFDB 3.0 (http://bioinfo.life.hust.edu.cn/AnimalTFDB2/) (10), which contains the information of transcription factors, transcriptional co-factors and chromatin remodelling factors. Furthermore, these response TFs were assigned with Boolean value 1 and −1, which represented up-regulation and down-regulation after perturbation, respectively. The gene symbols of mouse and rat were converted to human orthologue gene symbols with the Biomart R package (v2.38.0) (11) in order to combine the datasets from the three species. This operation was conducted, as the publicly available mammalian perturbation datasets mainly focus on these three species and the distribution of datasets among them is unbalanced. The gene expression profile of each dataset before perturbation was denoted as an initial gene expression profile (Figure 1A). In addition, the direct signalling protein targets of perturbagens were retrieved from Drug Repurposing Hub (www.broadinstitute.org/repurposing) (12), DrugBank (www.drugbank.ca) (13), and STITCH v5.0 (http://stitch.embl.de) (14) (Figure 1A). In STITCH, only the targets with a confidence value larger than 0.4 were kept along with the experiment and database evidence. The receptor targets of protein ligands were identified from manually curated ligand-receptor pairs from Ramilowski *et al.* (15). The effects of perturbagens on protein targets, activation, inhibition and unknown, were assigned with value 1, −1 and 2, respectively. When the reported effect was inconsistent between the databases, the effect was treated as unknown if any two databases reported contradictory effects (e.g.one database reported inhibition, another reported activation) or all databases reported unknown. Otherwise, we kept the effect as inhibition or activation if at least one database reported so and the other two were either consistent or unknown.

### Prediction of perturbation response TFs

The ChemPert tool for the prediction of response TFs after a query perturbation consists of three major steps (Figure 1B). In short, it first identifies TF response datasets perturbed with similar perturbagens as the query perturbagen. Then, it filters out the TF response datasets whose initial cell states are not similar to the query initial cell state. Finally, TFs are ranked by their frequencies of occurrence in the retrieved datasets. Thus, the output of this algorithm is a consensus response across multiple reference datasets selected based on the perturbagen similarity and initial cell state similarity that does not rely on prior cell annotations. We did not set any similarity threshold for the perturbation duration and concentration since the best result was obtained during our optimization of these parameters. The algorithmic details are described below.

Step 1: A modified Jaccard similarity between a query perturbagen and reference perturbagens in the ChemPert database is computed by:

$$\begin{equation*} J \left( {Q,R} \right) = \frac{{{{\left| {Q \cap R} \right|}}_{sign\ known} + {{\left| {Q \cap R} \right|}}_{sign\ unknown}}}{{\left| {Q \cup R} \right|}} \end{equation*}$$

where $Q$ is the target proteins of the query perturbagen and $R$ is the target proteins of the reference perturbagen being considered, ${| {Q \cap R} |}_{sign{\rm{\ }}known}{\rm{\ }}$is the cardinality of common protein targets (i.e. proteins that are targeted by both query perturbagen and reference perturbagen) with the same effect (activation or inhibition) between the query and reference perturbagens, whereas${\rm{\ }}{| {Q \cap R} |}_{sign{\rm{\ }}unknown}$ is the same cardinality computed among protein targets whose effects are unknown for the query and/or reference perturbagens. For the latter cardinality, a query protein target and a reference protein target are considered as a match regardless of their effects (activation or inhibition). Reference perturbagens with the modified Jaccard similarity higher than 1.5 z-score are retained. Then, all reference datasets perturbed by the retained perturbagens are retrieved from the ChemPert database.

Step 2: As perturbagen similarity between the query and reference perturbagens alone does not take into account the signalling state of the query cell type, which is important for determining the response profile, the algorithm addresses this issue by identifying signalling pathways that are likely active or permissive to perturbations. We reasoned that if the state of molecular paths from proteins targeted by a perturbagen to TFs is similar between the query and reference datasets, the TF response of the query data will also be similar to the reference response TFs. To compute such similarity, the prior knowledge network (PKN) is constructed by merging ReactomeFI (16), Omnipath (17) and DoRothEA v2 (18). Then, the short paths from one signalling protein to each downstream TF are identified as follows: first, the shortest path lengths from each signalling protein to all downstream TFs are calculated using the unweighted breadth-first algorithm implemented in R package igraph. Subsequently, the path length that can reach the largest number of downstream TFs from that signalling protein is considered as the maximum path length. We then calculate all possible short paths between the signalling proteins and all downstream TFs that are within this maximum path length. This procedure is repeated for every signalling protein in the PKN. Then, for each signalling protein–TF pair, a path enrichment analysis is performed using Fisher's exact test:

$$\begin{equation*}p = \frac{{\left( {a + b} \right)!\left( {c + d} \right)!\left( {a + c} \right)!\left( {b + d} \right)!}}{{a!b!c!d!n!}}\ \end{equation*}$$

where $a$ is the sum of normalized gene expression values of proteins present in all the short paths including the starting signalling protein and target downstream TF, $b$ is the sum of normalized gene expression values of all genes in the dataset, $c$ is the number of proteins present in all the short paths, $d$ is the total number of genes in the dataset, and $n$ is the sum of $a$, $b$, $c$ and $d$. The gene expression is normalized by the highest expression value in the dataset. Since Fisher's exact test can accept only integer values, the decimal values are rounded for $b$ and $a$.The *P*-values are corrected by the Benjamini–Hochberg method and paths with the adjusted *P*-value ≤0.05 are considered enriched. The initial cell state similarity between a query and a reference dataset is computed by the Jaccard similarity of common enriched paths. Reference datasets with this Jaccard similarity higher than *z*-score 1.5 are retained for the next step. The *z*-score is defined as:

$$\begin{equation*}Z = \frac{{x - \mu }}{\sigma }\ \end{equation*}$$

where $x$ in this case is a Jaccard similarity of a reference perturbagen w.r.t. the query perturbagen, and μ and σ are the mean and standard deviation of all reference perturbagens' Jaccard similarities w.r.t. the query perturbagen.

Step 3: The frequency of each response TF is computed among the reference datasets retained after Step 2. When a TF has both directions (i.e. up- or down-regulated), the one with the lower frequency is discarded. If this frequency is the same, the TF is discarded due to the uncertainty of its direction. Thus, the final output contains predicted response TFs in one direction and their frequency in the retained reference datasets. The frequency was also used for determining the ranking of predicted TFs (i.e. the more frequent, the higher). When a TF was not predicted, the 2067th rank was assigned to that TF, which is the number of TFs considered in ChemPert.

### Prediction of perturbagens targeting query TFs

Given a set of query TFs, ChemPert is also available for the prediction of perturbagens. The tool first identifies the potential signalling protein targets from the ChemPert database whose perturbation can induce a similar set of response TFs. Then, the perturbagens whose protein targets are enriched among the predicted signalling proteins are further identified (Figure 1B). This two-step approach enables us to predict both signalling proteins including surface receptors and protein ligands, and perturbagens such as small molecules and drugs. Moreover, this approach allows us to predict novel perturbagens that do not exist in the reference perturbation transcriptomics dataset. The similarity between query TFs and response TFs of each reference dataset in the ChemPert database is calculated by using a modified Jaccard similarity as:

$$\begin{equation*}J \left( {Q,R} \right) = \frac{{\mathop \sum \nolimits_{i = 1}^{\left| {Q \cap R} \right|} I\left( {{Q}_i,{R}_i} \right)}}{{\left| {Q \cup R} \right|}}\ \end{equation*}$$

with indicator function:

$$\begin{equation*}I \left( {{Q}_i,{R}_i} \right) = \left\{ \begin{array}{@{}*{1}{c}@{}} {1,if{Q}_i*{R}_i = 1}\\ {0,if{Q}_i*{R}_i = - 1} \end{array}\right. \end{equation*}$$

where $Q$ is the set of query TFs and $R$ is the response TFs for each reference in the ChemPert database. In order to ensure the consistent effect of a TF between the query and the reference, we modified the Jaccard similarity by adding an indicator function. If the TF has the same effect (both inhibition/activation), then 1 is assigned, and 0 otherwise. The perturbagens of the reference datasets are ranked based on the similarity in descending order. Only the highly confident perturbagens with z-score of similarity larger than 3.5 are selected for the further analysis. Next, ChemPert retrieves the signalling protein targets of each selected perturbagen from the ChemPert database and order the signalling proteins based on the sum of the similarity score of their corresponding perturbagens. The effects of signalling proteins are reported based on the majority effect of their perturbagens. For example, value 1 is assigned to the signalling protein when more predicted perturbagens have activation effect on it. The signalling protein is assigned as 2 when all of its predicted perturbagens have unknown effect on it.

Finally, the prediction of perturbagens is conducted as follows: each perturbagen and corresponding protein targets in ChemPert database is converted into a regulon-like class as TF-regulons in database DoRothEA v2. Then, we carried out analytic rank-based enrichment analysis (aREA) implemented in the VIPER R package v1.18.1 (19), which takes advantage of TF-regulon interactions for identifying TFs that are enriched for the regulon targets. Here, we replaced TF-regulons with our perturbagen-target regulon-like class to predict perturbagens. By doing so, we aim to identify the perturbagens whose protein targets were enriched among the top ranked predicted signalling proteins. We use top 500 predicted signalling proteins for this step. The predicted perturbagens are ranked based on the normalized enriched score (NES) and the ones with false discovery rate less than 0.05 are kept.

### Evaluation of ChemPert database

The predictive performance of the ChemPert database was compared to a cancer database using the subset of the LINCS L1000 database, which only contains cancer cell datasets (2). We performed a leave-one-out validation, in which one reference dataset in the ChemPert database was randomly selected as a query dataset and removed from database. This query dataset was used to compare the performance between using the ChemPert database and using the cancer database in terms of response TFs prediction and perturbagens prediction. This validation was performed by randomly selecting 4000 datasets and this procedure was repeated 10 times. In addition, the difference in transcriptional responses between non-cancer cells and cancer cells was quantified using perturbagens that are commonly used for at least three cell types in both ChemPert and cancer database. The Jaccard similarity of transcriptional responses within non-cancer cells (within-ness) and that between non-cancer and cancer cells (between-ness) were calculated and compared. The perturbagens whose within-ness are significantly larger than the between-ness were identified by using one-side Wilcoxon test with adjust *P*-value <0.05.

### GSEA and QuaternaryProd

Reactome (20), Gene Ontology Biological Process (GOBP) (21) and WikiPathway (22) were download from the EnrichR web site (23). QuaternaryProd (24) was run using the causal relation engine with Quaternary Dot Product scoring statistic over the human STRINGdb, as suggested by the authors. Gene symbols for the mouse datasets are converted into human homologous Entrez IDs. The default parameter values were used, but the log fold change threshold log_2_ (1.5) was used to ensure the agreement with the DEGs for the ChemPert database. Since QuaternaryProd predicts only signalling proteins, the ChemPert algorithm for the prediction of perturbagens was applied to identify perturbagens targeting the predicted signalling proteins. As QuaternaryProd required datasets with at least two replicates for both before and after perturbation samples, datasets with less than two replicates were discarded.

### Construction of ChemPert web interface

The ChemPert web interface was implemented using Python 3.7 (https://www.python.org/) programming language and constructed using the Django (https://www.djangoproject.com/), a high-level Python web framework. In the Django web framework, the front-end responsive web pages were built using the HTML templates combined with Semantic UI (https://semantic-ui.com/) and Bootstrap (https://getbootstrap.com/) libraries. The responsive table widget with filter, search and pagination functionalities in some web pages was implemented using django-filter (https://django-filter.readthedocs.io/) and django-tables2 (http://django-tables2.readthedocs.io/) libraries. The Django framework provides data-model syntax, the data is defined in the Django model and is easily mapped to the SQLite Database (https://www.sqlite.org/index.html). Finally, this web project was hosted on a Rocky Linux 8 (https://rockylinux.org/) server.

## RESULTS

### Composition of ChemPert database

**Figure 2.**
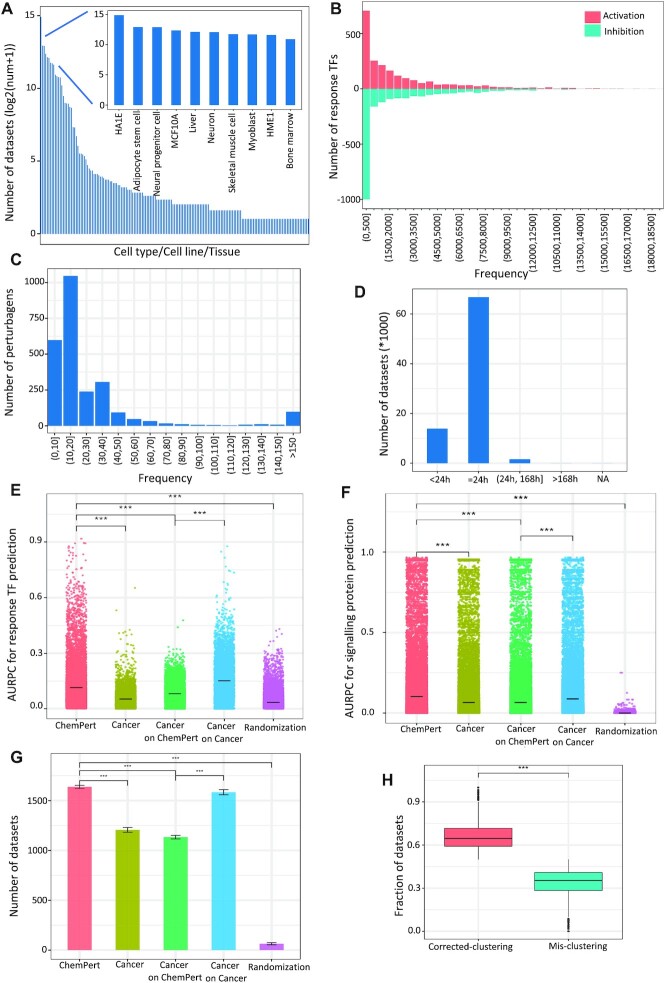
The compositions and evaluation of ChemPert database. (**A**) Distribution of datasets across different cell types/lines/tissues in the ChemPert database. Y-axis scale is log_2_(number + 1) for each cell type/line/tissue. (**B**) Frequency of TFs in the ChemPert database, including inhibited and activated ones. X-axis represents the frequency of TFs and y-axis presents the number of TFs with corresponding frequency. (**C**) Distribution of perturbagen frequency in the ChemPert database. X-axis represents the frequency of perturbagen, and y-axis represents the number of perturbagen with corresponding frequency. (**D**) Distribution of datasets for different perturbation durations. (**E**) AURPC for response TF prediction given perturbagens. (**F**) AURPC for protein target prediction given response TFs. (**G**) Number of datasets with correct perturbagen prediction, data are mean ± MSE. E–G used the benchmarking datasets to compare the performance of ChemPert tool using the ChemPert database, cancer database or randomization. Significance was calculated by using one-sided Wilcoxon test. ***: *P*-value < 2.22e−16. (**H**) Fraction of perturbagens whose within-ness are significantly larger than between-ness.

In order to infer the relationship between the signalling perturbation and downstream transcriptional responses, we exhaustively collected and compiled transcriptome profiles of chemical perturbations applied solely on non-cancer cells from public resources (see Materials and Methods). This resulted in a database consisting of 82 270 transcriptional signatures derived from 2566 unique perturbagens across 167 unique normal cell types/lines/tissues (Figure 2A). The datasets covered 2132 unique TFs, in both activation (up) and inhibition (down) directions with no significant bias towards either of them (Figure 2B). The breakdown of the DETFs by species is shown in Supplementary Figure S1. More than half of the perturbagens (∼65%) have frequency not larger than 20 (Figure 2C) and majority of the perturbagens (∼98%) in the ChemPert database have duration not larger than 24 h (Figure 2D). In addition, we also collected and integrated the protein targets and corresponding effects (activation, inhibition or unknown) of 57 818 chemical compounds.

### Benchmarking of ChemPert

The mapping between signalling perturbations and response TFs enables *in silico* predictions of either the downstream effects of given perturbagens or the perturbagens that can target given sets of TFs. In particular, such mapping for non-cancer cells will significantly reduce our efforts for identifying perturbagens of desired effects instead of the perturbagens killing cells in cancer therapies, which will aid in a wide range of biological and clinical applications. Therefore, we developed a computational tool for either predicting downstream response TFs given a perturbagen of known target proteins, or the perturbagens of desired TF responses.

To evaluate the importance of using the ChemPert database, rather than cancer cell databases, for the prediction in non-cancer cell types, we conducted a benchmark analysis on the ChemPert database and on the cancer database solely consisting of cancer perturbation datasets (see Materials and Methods). The results show a significantly higher performance (measured as the area under precision-recall curve (AUPRC)) with the ChemPert database than with the cancer database in the prediction of response TFs (Figure 2E, ‘ChemPert’ and ‘Cancer’). In fact, the performance of the latter was similar to the random selection of reference datasets (Figure 2E, ‘Randomization’). We also investigated if a similar predictive performance could be achieved without taking into account the initial cell states (i.e. based only on perturbagen target similarities). This result shows a significant decrease in the performance (Supplementary Figure S2A), indicating that perturbagen similarity alone is not sufficient for mapping cell-specific response TFs. In accordance with this, the rank of TF hits was also significantly worse when the initial cell states were not considered (Supplementary Figure S2B). As for the prediction of perturbagens from response TFs, the AUPRC of signalling protein targets was significantly, albeit slightly, better when using the ChemPert database compared to using the cancer database (Figure 2F, ‘ChemPert’, ‘Cancer’). Moreover, using the ChemPert database significantly increased the number of datasets with true perturbagen prediction (Figure 2G, ‘ChemPert’, ‘Cancer’) and the rank of true perturbagens was significantly lower (Supplementary Figure S2B, ‘ChemPert’, ‘Cancer’).

Next, we wondered whether the observed increase in the predictive performance was due to the higher number of unique perturbagens in the non-cancer database (2551) than the cancer database (2198) rather to the unsuitability of cancer cells for making predictions for non-cancer cells. To this end, first the number of signalling pathways targeted by these perturbagens was examined using the Reactome database. Of the 1530 Reactome signalling pathways, 1461 are targeted at least once by the perturbagens in the non-cancer database, whereas 1425 are targeted at least once by the perturbagens in the cancer database, which leaves only 36 pathways that are not covered by the cancer database. Then, in order to assess the significance of the reference database, we applied our algorithm to make predictions for the cancer datasets using either the non-cancer database or the cancer database. The result showed that the performance significantly dropped when the non-cancer database was used in comparison to when the cancer database was used (Figure 2E-G, Supplementary Figure S2B, ‘Cancer on ChemPert’ and ‘Cancer on Cancer’, respectively). Furthermore, the performance was also significantly worse than that for the non-cancer predictions (Figure 2E-G, Supplementary Figure S2B, ‘Cancer on ChemPert’ and ‘ChemPert’, respectively), indicating that the cancer database can give better predictions for cancer cells than the non-cancer database and that the increased performance for non-cancer cells based on the non-cancer database is not due to the higher number of unique perturbagens in the database but rather due to the higher similarity in response TF profiles. To further investigate the effect of the cancer database on predictions for non-cancer cells, we performed the same benchmarking to examine whether combining the non-cancer database and the cancer database could improve the predictive accuracy for non-cancer cells. However, this operation slightly but significantly decreased the overall performance in both response TF prediction and signalling protein or perturbagen prediction (Supplementary Figure S3A–E). Indeed, a closer examination of the cases where the performance significantly decreased when the cancer database was added revealed that the response TF profiles of non-cancer and cancer cells largely formed two distinct clusters (Supplementary Figure S4) even when the origin of cells was the same (e.g. healthy hepatocyte and HEPG2 cell line). Overall, the clustering of response TF profiles between normal and cancer cells upon 1569 unique perturbations in the database indicated that the fraction of cells correctly clustered to their respective class (i.e. non-cancer or cancer) was significantly higher than mis-clustered ones (one-sided Wilcoxon test, *P*-value < 2.22e−16) (Figure 2H). These results indicate that the cancer database will add noise to reponse TF prediction of a query perturbagen, giving an explanation for why using the cancer database is detrimental for the response TF prediction in non-cancer cells. A significant decrease in signalling protein / perturbagen prediction can also be explained by the confounding effect of cancer datasets. For example, tranylcypromine, a commonly used drug for the treatment of depression, was predicted for neural progenitor cells (NPC.TAK) by using the non-cancer database while not predicted by using both non-cancer and cancer databases. The hierarchical clustering revealed that the response TF profile of this cell type had a higher similarity to those of other non-cancer cell types than to those of cancer cell types (Supplementary Figure S5A). However, the response TF profile of NPC.TAK cells to tranylcypromine also had high similarities to cancer cells that were perturbed with different perturbagens (Supplementary Figure S5B). This confounding effect of cancer cells led to the failure of the algorithm to find the correct perturbagen.

Taken together, our benchmarking results highlight the importance of use of non-cancer cell perturbation database for mapping between signalling perturbations and response TFs in non-cancer cells. The results also support our notion that cancer cells are not optimal for this objective due presumably to their significantly altered signalling and transcriptional logics that result in distinct TF responses.

### Benchmarking with GSEA-based approaches

We compared our algorithm to more widely employed GSEA-based signalling pathway inference approaches. The most common input gene set for GSEA is DEGs, however, they are not available for response TF prediction. Therefore, we first performed GSEA for signalling pathways that are enriched in the initial cell state using the same approach described in Step 2 of our response TF prediction algorithm and then further identified pathways that are targetted by the query perturbagen. Reactome, Gene Ontology Biological Process (GOBP) and WikiPathway were used for this analysis since these are most widely used for pathway GSEA. Finally, the presence of correct response TFs in these signalling pathways was counted and the algorithmic performance was quantified by the AUPRC. For the prediction of signalling proteins/perturbagens, we used DEGs between before- and after perturbations as input to GSEA using the EnrichR R package for the same three pathway databases. In addition, QuaternaryProd was also used, which, given a set of DEGs, identifies upstream signalling proteins by performing causal reasoning with a statistical test based on networks. Then, we ranked signalling proteins by their frequencies of appearance in the enriched pathways. Finally, perturbagen prediction was carried out based on these predicted signalling pathways using our algorithm. The result showed that GSEA is not as accurate as our algorithm in predicting both response TFs and signalling proteins regardless of the used pathway database (Supplementary Figure S6A, B). Accordingly, the perturbagen prediction was also significantly better for our algorithm than the other approaches (Supplementary Figure S6C). In summary, ChemPert outperforms GSEA-based pathway inference approaches in both response TF prediction and perturbagen prediction.

### Description of ChemPert web interface

**Figure 3.**
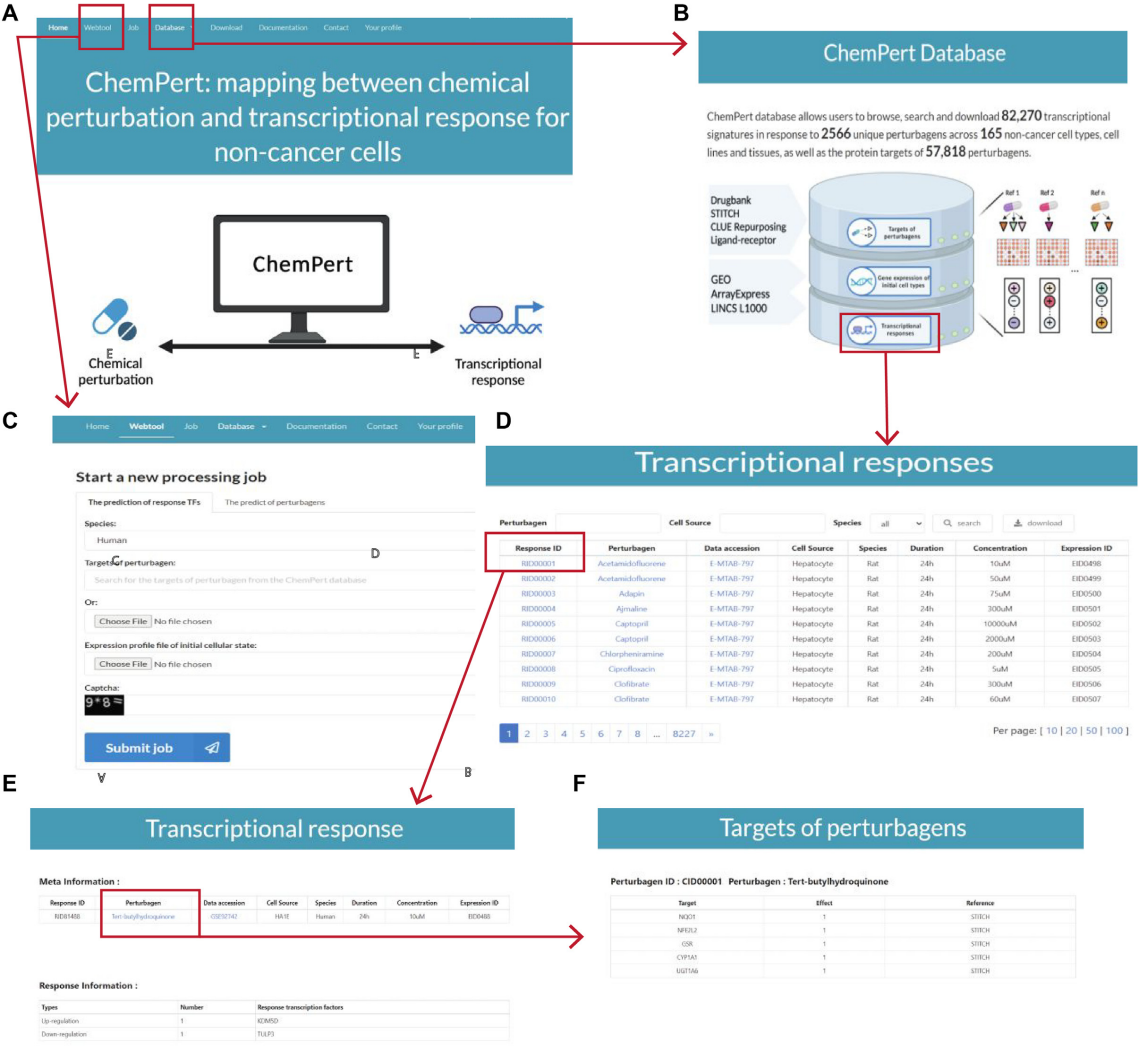
Illustration of ChemPert web interface. (**A**) The home page of web interface. ChemPert mainly consists of two sections: database and webtool. (**B**) The home page of the ChemPert database. The database is composed of three parts: targets of perturbagens, gene expression of initial cell types and transcriptional response. Clicking the button can switch to corresponding part. (**C**) The webtool page. Users can predict either the response TFs of given perturbagen or the perturbagens targeting desired query TFs. (**D**) The transcriptional response table listing the meta information of datasets. (**E**) Detailed transcriptional response for one dataset. (**F**) Information about targets of perturbagens.

The ChemPert web interface mainly includes two sections (Figure 3A): the database (Figure 3B) and the webtool (Figure 3C). The database section allows users to browse, search and download any datasets in ChemPert without creating an account and login. The home page of the database section provides a summary of the database and allows users to get access to one of the three main resources of the databases, the targets of perturbagens, the gene expression profiles of initial cellular states and the TF responses after perturbations (Figure 3B). For example, when users click the button ‘Transcriptional responses’, a table listing the major meta information on each dataset will be returned, including the perturbagen, data accession number, cell type, perturbation duration and concentration (Figure 3D). The search area allows users to search for the datasets of interest based on the perturbagens, cell types or species (Figure 3D). In particular, users can click the ‘Response ID’ to browse the response TFs of corresponding dataset (Figure 3E). Clicking the ‘Perturbagen’ button enables the users to browse the protein targets of this chemical compound (Figure 3F). In addition, users can download the datasets of interest or download all datasets from ‘Download’ page.

The webtool section provides an intuitive interface for users to predict either response TFs or perturbagens (Figure 3C). To predict response TFs of a query perturbagen, users can search for the targets of perturbagen in the ChemPert database as input. If a query perturbagen is not available in the database for the prediction of response TFs, users can still run the tool by providing the protein targets of the query perturbagen as input. Users will be informed by email and subsequently download the results through the link in the email when the job is done. The response TF prediction tool takes between 2.5–3 hours with four CPUs depending on users internet connection speed. Currently, the web server has only four CPUs and the tool can be run once at a time. The perturbagen prediction tool takes roughly 2–5 min with four CPUs. The detailed usage of ChemPert web interface is described in ‘Documentation’ page.

### Use case - ChemPert predicts cell state-specific responses to drugs in non-alcoholic steatohepatitis (NASH)

NASH is an advanced form of non-alcoholic fatty liver disease (NAFLD) that not only causes the accumulation of fat in the liver but also inflammation and damage to liver cells. This can cause scarring, cirrhosis and even liver cancer and can be lethal, but currently no FDA-approved medications exist (25,26). We applied ChemPert to the RNA-seq data of two models of diet-induced NASH to predict the TF responses of perturbagens that could enable us to find optimal treatments. The first model consists of mice fed with a high-fat diet rich in fructose, palmitate, and cholesterol (FPC diet) for 20 weeks (27). The second model consists of mice fed with a choline-deficient, methionine-reduced (CDA) high-fat diet for seven weeks (28). In addition, both models were stratified into two groups based on the severity of the liver disease phenotype: mild NASH and advanced NASH. Mice with advanced NASH had significantly more inflammatory foci and collagen fiber formation compared to mice with mild NASH (29). The use of both diet models and their two disease severity phenotypes allows us to take advantage of the heterogeneous NASH states and make more reliable assessment of predicted response TFs, as an effective drug for the treatment of NAFLD must be effective at different stages. ChemPert was run for three perturbagens: obeticholic acid (OCA) known to significantly improve fibrosis in adult patients with definite NASH (30); pioglitazone and vitamin E, associated with reductions in hepatic steatosis and lobular inflammation, but with no improvement in fibrosis score (31).

**Figure 4.**
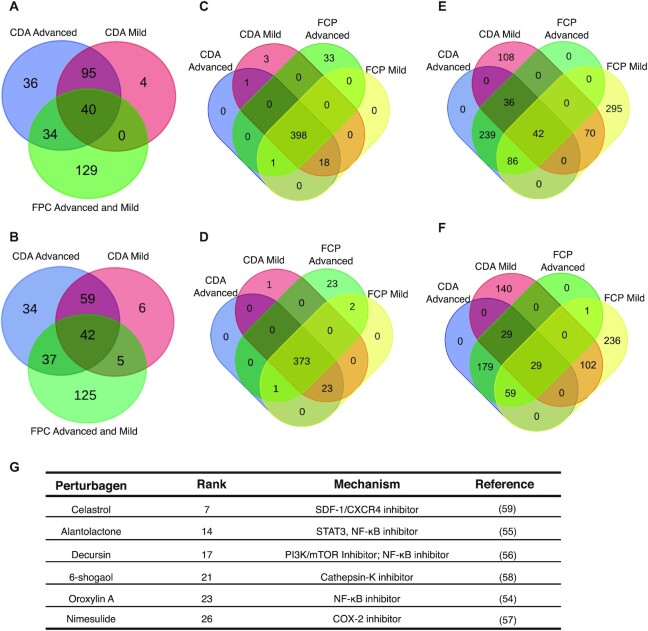
Application of ChemPert. (A–F) Venn diagrams showing overlaps of predicted TFs among different diets and disease states of NASH models. Up-regulated TFs (**A**) and down-regulated TFs (**B**) after OCA perturbation, up-regulated TFs (**C**) and down-regulated TFs (**D**) after pioglitazone perturbation, up-regulated TFs (**E**) and down-regulated TFs (**F**) after vitamin E perturbation. (**G**) The representative of predicted perturbagens with literatures support for the treatment of OA. Details are shown in Supplementary Table S4.

In the case of OCA, 209 TFs were predicted to be upregulated in the CDA model, 135 of which were predicted to be overexpressed in both mild and severe models (Figure 4A). In the FPC model, upregulation of 203 TFs in response to OCA was predicted regardless of disease severity. Among all these TFs, 40 were common in both NASH models. Due to the low number of common TFs, the GSEA analysis did not identify any enriched pathway. However, consistent with the recognized therapeutic effect of OCA, these common TFs are related not only to hepatic steatosis and steatohepatitis improvements (ATF6, HBP1, BTG1, SAP18, PPARD, PPARG, BIRC2), but also to anti-fibrotic effects (FOXO1, INSR, KLF6) and blocking of disease progression (DACH1, RYBP, ZFP36L1). Similarly, the 42 common downregulated TFs (Figure 4B) include both signatures of steatosis and obesity (CNOT3, CREB3L3, REPIN1, STAT1), and signatures of fibrosis (CCNE1, ETS1, HDAC6, HDAC9, HLF, PLAGL1, SOX4, TRIM16, TRIM29) and hepatocellular carcinoma (HCC) (BCL3, MYCBP, SMARCA4). The detailed explanation for each TF can be found in Supplementary Note.

The pioglitazone perturbation predicted 421 and 449 total up-regulated TFs in the CDA and FPC models, respectively, 398 of which are common to both disease models and disease states (Figure 4C). The GSEA of these 398 TFs (Supplementary Table S1) contained Nuclear Receptor transcription pathway including PPARD and PPARG, as expected, since the thiazolidinediones, such as pioglitazone, are synthetic agonists for these receptors, that play a key role in lipid metabolism. However, the GSEA also produced TGF-b signalling which is a well-known profibrogenic cytokine due to its role in hepatic stellate cell (HSC) activation and extracellular matrix production. This pathway has been described to contribute to all stages of liver disease progression, from initial liver injury through inflammation and fibrosis to cirrhosis and hepatocellular carcinoma (HCC) (32–34). Moreover, TRAF6 Mediated Induction of proinflammatory cytokines is a key driving force of proinflammatory and profibrogenic responses in NASH (35) and has been described as a possible contributor to progression to HCC (36). TLR4 signalling repertoire is involved in a variety of liver injury including that induced by NASH, which has been shown to play a key role during fibrogenesis in preclinical models of NAFLD (37), as wells as to enhance TGF-β signalling (38). Stabilization of p53 has also been involved in the pathogenesis of fatty liver disease (39). On the other hand, the GSEA of 376 common down-regulated TFs (Figure 4D, Supplementary Table S2) included the Interferon gamma (IFN-γ) signalling, which has previously shown promising results in terms of fibrosis scores in patients with chronic HBV infection, most likely by antagonizing profibrogenic transforming TFG-β effects (40); and in accordance with these data, a preclinical IFN-γ deficient model showed a rapid development of liver fibrosis when fed a fatty diet (41).

Finally, the vitamin E perturbation obtained 581 and 768 total upregulated TFs for the CDA and FPC models, respectively, 42 of which are common to both disease models and disease states (Figure 4E). The GSEA of these TFs (Supplementary Table S3) identified, as in Pioglitazone, the Nuclear Receptor transcription pathway, but also the Regulation of Lipid Metabolism by Peroxisome proliferator-activated receptor alpha (PPAR- a). Furthermore, the Toll Like Receptor 3 (TLR3) Cascade and TRIF mediated TLR3 signalling were enriched. Activation of TLR3 in HSCs has been demonstrated to exacerbate liver fibrosis (42). The GSEA of 29 common down-regulated TFs (Figure 4F) did not result in any enrichment. However, these TFs include FOXO1 and KLF6, which identified as anti-fibrotic (43–45) that were predicted to be upregulated in the OCA perturbation. Others are ID2, which reduces differentiation of HSCs and thus inhibits liver fibrosis (46), RUNX1, which regulates the expression of angiogenic and adhesion molecules, enhancing inflammation and disease severity in NASH (47), and KLF2, which has been reported to be elevated in livers from obese mice, and to induce triglycerides accumulation (48).

Overall, the analysis with OCA predicted the up-regulation of TFs related to the inhibition of HSC activation responsible for the collagen deposition in liver tissue during fibrogenesis (49), along with TFs described as protective against inflammatory response and hepatic fat deposition, and down-regulation of TF signatures of steatosis, fibrosis and HCC. Although the common TFs of pioglitazone and vitamin E perturbations appeared to be viable for treating hepatic steatosis and inflammation, none of these were associated with improvement of fibrosis. Thus, this analysis demonstrates that ChemPert is valid for predicting the transcriptional effects of different drugs at different stages of NAFLD and could be a useful tool for pre-screening a wide range of chemical treatments prior to the pre-clinical or clinical studies.

### Use case—ChemPert predicts novel perturbagens for the treatment of osteoarthritis (OA) and NASH

OA is a complex degenerative disease leading to disability and characterized by cartilage degradation, synovial inflammation, and bone remodelling (50). Currently, effective pharmacologic therapies for OA are still not available and more specific approaches are desirable (51). Thus, ChemPert was applied to OA to investigate potential therapeutic treatments. The differentially expressed TFs in human osteoarthritis cartilage compared to non-osteoarthritis individuals were identified as input (GSE169077). A considerable number of known clinical or pre-clinical chemical compounds for the treatment of OA were recapitulated by ChemPert (Figure 4G, Supplementary Table S4). The nuclear factor-kappaB (NF-κB) signalling pathway is regarded as potential targets for the therapeutic treatment of OA, since NF-κB is aberrantly upregulated in OA patients and NF-κB is included in many OA-associated events, including chondrocyte catabolism, chondrocyte survival, and synovial inflammation (52,53). In agreement with this, several perturbagens targeting NF-kB were predicted by ChemPert, including oroxylin A (54), alantolactone (55) and decursin (56), which all have been shown to ameliorate OA. These perturbagens attenuate OA progression by inhibition of inflammatory response, hypertrophy, cartilage degeneration or impaired autophagy triggered by IL-1β. Moreover, ChemPert also predicted the perturbagen, nimesulide, a cyclo-oxygenase (COX)-2-selective inhibitor that attenuates the pain associated with walking for OA patients (57). The prediction 6-shogaol has been shown to significantly reduce the hypertrophic markers in cartilage and prevent synovial inflammation and cartilage degradation in OA (58). Celastrol was also predicted, which is known to target SDF-1/CXCR4 signalling pathway is able to attenuate pain and cartilage damage in OA (59) and has the potential to prevent OA by inhibiting the ERs-mediated apoptosis (57). Studies also revealed that the PI3K/AKT/mTOR pathway plays a crucial role in cartilage degradation and can be used as a therapeutic target for the clinical intervention of OA (60,61). Consistently, we identified the signalling proteins that are enriched in PI3K/AKT pathway (Supplementary Figure S7) and the perturbagens that inhibit the PI3K/AKT signalling pathway, including oroxylin A (62), KU-0063794 (63), and other novel perturbagens such as NVP-BEZ235 and TG100-115 (Supplementary Table S4). In addition, previous reports have indicated that VEGF can be a biomarker for patients with OA, which is highly expressed in articular cartilage, synovium, subchondral bone and serum of OA patients (64). Indeed, we identified the signalling proteins that are enriched in VEGF pathway and predicted corresponding inhibitors, like WHI-P180 and PP-121. Furthermore, another novel prediction is 1,5-isoquinolinediol, a PARP-1 inhibitor. In accordance with our prediction, a previous study also reported that PARP-1 inhibitors are able to decrease the inflammatory response in the cartilage of OA rat model (65). Finally, we applied the algorithm also to the sane four mouse models of NASH used in the previous section (i.e. FPC Mild, FPC Adv, CDA Mild and CDA Adv) to predict novel perturbagens for NASH treatment using the DETFs between the control and each of the four models. This analysis predicted 93 perturbagens common to all the four models and 59 common to both advanced NASH models (Supplementary Figure S8), many of which have been implicated in the amelioration of the progression of steatohepatitis, fibrosis and hepatocarcinoma. The detailed discussion of individual predicted perturbagens can be found in Supplementary Note.

To summarize, ChemPert not only recapitulated the known perturbagens, but also provided novel predictions as potential therapies for the treatment of OA. These results demonstrate the usability of ChemPert for *in silico* chemical screening and drug discovery, and can be generally applicable to different diseases to prioritize the perturbagens that reverse the disease phenotypes to the healthy counterparts.

## DISCUSSION

ChemPert is the first comprehensive compendium of manually curated perturbation transcriptomics exclusively for non-cancer cells, providing a valuable resource for both experimental researchers who wish to find datasets relevant to their research, but also computational researchers who need a non-cancer perturbation transcriptomics dataset for developing novel algorithms. In addition, ChemPert provides a computational tool that leverages the non-cancer cell data to predict either TF responses after perturbations, or perturbagens that target desired sets of TFs. Importantly, predictions generated for non-cancer cells when using ChemPert database were significantly more accurate than those based on cancer databases. Due to the scarcity of available combinatorial perturbation datasets, we focus on transcriptional signatures of single-agent perturbations in the current version of ChemPert. However, our future plan is to continue adding new non-cancer combinatorial perturbation datasets to address the important challenge of *in silico* combinatorial drug screening. In addition, we will regularly collect and compile new single-agent perturbation datasets to maintain the state-of-the-art of the database.

## DATA AVAILABILITY

The ChemPert web interface is freely accessible at: https://chempert.uni.lu/. ChemPert was implemented in R and is available from Gitlab (https://git-r3lab.uni.lu/CBG/chempert).

## Supplementary Material

gkac862_Supplemental_Files

## References

[1] Lamb J. , Crawford E.D., Peck D., Modell J.W., Blat I.C., Wrobel M.J., Lerner J., Brunet J.P., Subramanian A., Ross K.N. et al. The connectivity map: using gene-expression signatures to connect small molecules, genes, and disease. Science. 2006; 313:1929–1935. 17008526 10.1126/science.1132939

[2] Subramanian A. , Narayan R., Corsello S.M., Peck D.D., Natoli T.E., Lu X., Gould J., Davis J.F., Tubelli A.A., Asiedu J.K. et al. A next generation connectivity map: L1000 platform and the first 1,000,000 profiles. Cell. 2017; 171:1437–1452. 29195078 10.1016/j.cell.2017.10.049 PMC5990023

[3] Wang Z. , Clark N.R., Ma’ayan A. Drug-induced adverse events prediction with the LINCS L1000 data. Bioinformatics. 2016; 32:2338–2345. 27153606 10.1093/bioinformatics/btw168 PMC4965635

[4] Wang Y.Y. , Kang H., Xu T., Hao L., Bao Y., Jia P. CeDR atlas: a knowledgebase of cellular drug response. Nucleic Acids Res. 2022; 50:D1164–D1171. 34634794 10.1093/nar/gkab897 PMC8728137

[5] Napolitano F. , Rapakoulia T., Annunziata P., Hasegawa A., Cardon M., Napolitano S., Vaccaro L., Iuliano A., Wanderlingh L.G., Kasukawa T. et al. Automatic identification of small molecules that promote cell conversion and reprogramming. Stem Cell Rep. 2021; 16:1381–1390. 10.1016/j.stemcr.2021.03.028 PMC8185468 33891873

[6] Sharma S. , Petsalaki E. Large-scale datasets uncovering cell signalling networks in cancer: context matters. Curr. Opin. Genet. Dev. 2019; 54:118–124. 31200172 10.1016/j.gde.2019.05.001

[7] Barrett T. , Wilhite S.E., Ledoux P., Evangelista C., Kim I.F., Tomashevsky M., Marshall K.A., Phillippy K.H., Sherman P.M., Holko M. et al. NCBI GEO: archive for functional genomics data sets–update. Nucleic Acids Res. 2013; 41:D991–D995. 23193258 10.1093/nar/gks1193 PMC3531084

[8] QKolesnikov N. , Hastings E., Keays M., Melnichuk O., Tang Y.A., Williams E., Dylag M., Kurbatova N., Brandizi M., Burdett T. et al. ArrayExpress update–simplifying data submissions. Nucleic Acids Res. 2015; 43:D1113–D1116. 25361974 10.1093/nar/gku1057 PMC4383899

[9] Ritchie M.E. , Phipson B., Wu D., Hu Y., Law C.W., Shi W., Smyth G.K. limma powers differential expression analyses for RNA-sequencing and microarray studies. Nucleic Acids Res. 2015; 43:e47. 25605792 10.1093/nar/gkv007 PMC4402510

[10] Hu H. , Miao Y.R., Jia L.H., Yu Q.Y., Zhang Q., Guo A.Y. AnimalTFDB 3.0: a comprehensive resource for annotation and prediction of animal transcription factors. Nucleic Acids Res. 2019; 47:D33–D38. 30204897 10.1093/nar/gky822 PMC6323978

[11] Durinck S. , Spellman P.T., Birney E., Huber W. Mapping identifiers for the integration of genomic datasets with the R/Bioconductor package biomaRt. Nat. Protoc. 2009; 4:1184–1191. 19617889 10.1038/nprot.2009.97 PMC3159387

[12] Corsello S.M. , Bittker J.A., Liu Z., Gould J., McCarren P., Hirschman J.E., Johnston S.E., Vrcic A., Wong B., Khan M. et al. The drug repurposing hub: a next-generation drug library and information resource. Nat. Med. 2017; 23:405–408. 28388612 10.1038/nm.4306 PMC5568558

[13] Wishart D.S. , Feunang Y.D., Guo A.C., Lo E.J., Marcu A., Grant J.R., Sajed T., Johnson D., Li C., Sayeeda Z. et al. DrugBank 5.0: a major update to the drugbank database for 2018. Nucleic Acids Res. 2018; 46:D1074–D1082. 29126136 10.1093/nar/gkx1037 PMC5753335

[14] Szklarczyk D. , Santos A., von Mering C., Jensen L.J., Bork P., Kuhn M. STITCH 5: augmenting protein-chemical interaction networks with tissue and affinity data. Nucleic Acids Res. 2016; 44:D380–D384. 26590256 10.1093/nar/gkv1277 PMC4702904

[15] Ramilowski J.A. , Goldberg T., Harshbarger J., Kloppmann E., Lizio M., Satagopam V.P., Itoh M., Kawaji H., Carninci P., Rost B. et al. A draft network of ligand-receptor-mediated multicellular signalling in human. Nat. Commun. 2015; 6:7866. 26198319 10.1038/ncomms8866 PMC4525178

[16] Wu G. , Feng X., Stein L. A human functional protein interaction network and its application to cancer data analysis. Genome Biol. 2010; 11:R53. 20482850 10.1186/gb-2010-11-5-r53 PMC2898064

[17] Türei D. , Korcsmáros T., Saez-Rodriguez J. OmniPath: guidelines and gateway for literature-curated signaling pathway resources. Nat. Methods. 2016; 13:966–967. 27898060 10.1038/nmeth.4077

[18] Garcia-Alonso L. , Holland C.H., Ibrahim M.M., Turei D., Saez-Rodriguez J. Benchmark and integration of resources for the estimation of human transcription factor activities. Genome Res. 2019; 29:1363–1375. 31340985 10.1101/gr.240663.118 PMC6673718

[19] Alvarez M.J. , Shen Y., Giorgi F.M., Lachmann A., Ding B.B., Ye B.H., Califano A. Functional characterization of somatic mutations in cancer using network-based inference of protein activity. Nat. Genet. 2016; 48:838–847. 27322546 10.1038/ng.3593 PMC5040167

[20] Fabregat A. , Jupe S., Matthews L., Sidiropoulos K., Gillespie M., Garapati P., Haw R., Jassal B., Korninger F., May B. et al. The reactome pathway knowledgebase. Nucleic Acids Res. 2018; 46:D649–D655. 29145629 10.1093/nar/gkx1132 PMC5753187

[21] Ashburner M. , Ball C.A., Blake J.A., Botstein D., Butler H., Cherry J.M., Davis A.P., Dolinski K., Dwight S.S., Eppig J.T. et al. Gene ontology: tool for the unification of biology. The gene ontology consortium. Nat. Genet. 2000; 25:25–29. 10802651 10.1038/75556 PMC3037419

[22] Pico A.R. , Kelder T., van Iersel M.P., Hanspers K., Conklin B.R., Evelo C. WikiPathways: pathway editing for the people. PLoS Biol. 2008; 6:e184. 18651794 10.1371/journal.pbio.0060184 PMC2475545

[23] Kuleshov M.V. , Jones M.R., Rouillard A.D., Fernandez N.F., Duan Q., Wang Z., Koplev S., Jenkins S.L., Jagodnik K.M., Lachmann A. et al. Enrichr: a comprehensive gene set enrichment analysis web server 2016 update. Nucleic Acids Res. 2016; 44:W90–W97. 27141961 10.1093/nar/gkw377 PMC4987924

[24] Fakhry C.T. , Choudhary P., Gutteridge A., Sidders B., Chen P., Ziemek D., Zarringhalam K. Interpreting transcriptional changes using causal graphs: new methods and their practical utility on public networks. BMC Bioinf. 2016; 17:318. 10.1186/s12859-016-1181-8 PMC4995651 27553489

[25] Ando Y. , Jou J.H. Nonalcoholic fatty liver disease and recent guideline updates. Clin Liver Dis (Hoboken). 2021; 17:23–28. 33552482 10.1002/cld.1045 PMC7849298

[26] European Association for the Study of the Liver (EASL), European Association for the Study of Diabetes (EASD), European Association for the Study of Obesity (EASO) EASL-EASD-EASO clinical practice guidelines for the management of non-alcoholic fatty liver disease. J. Hepatol. 2016; 64:1388–1402. 27062661 10.1016/j.jhep.2015.11.004

[27] Wang X. , Zheng Z., Caviglia J.M., Corey K.E., Herfel T.M., Cai B., Masia R., Chung R.T., Lefkowitch J.H., Schwabe R.F. et al. Hepatocyte TAZ/WWTR1 promotes inflammation and fibrosis in nonalcoholic steatohepatitis. Cell Metab. 2016; 24:848–862. 28068223 10.1016/j.cmet.2016.09.016 PMC5226184

[28] Matsumoto M. , Hada N., Sakamaki Y., Uno A., Shiga T., Tanaka C., Ito T., Katsume A., Sudoh M. An improved mouse model that rapidly develops fibrosis in non-alcoholic steatohepatitis. Int. J. Exp. Pathol. 2013; 94:93–103. 23305254 10.1111/iep.12008 PMC3607137

[29] Loft A. , Alfaro A.J., Schmidt S.F., Pedersen F.B., Terkelsen M.K., Puglia M., Chow K.K., Feuchtinger A., Troullinaki M., Maida A. et al. Liver-fibrosis-activated transcriptional networks govern hepatocyte reprogramming and intra-hepatic communication. Cell Metab. 2021; 33:1685–1700. 34237252 10.1016/j.cmet.2021.06.005

[30] Younossi Z.M. , Ratziu V., Loomba R., Rinella M., Anstee Q.M., Goodman Z., Bedossa P., Geier A., Beckebaum S., Newsome P.N. et al. Obeticholic acid for the treatment of non-alcoholic steatohepatitis: interim analysis from a multicentre, randomised, placebo-controlled phase 3 trial. Lancet. 2019; 394:2184–2196. 31813633 10.1016/S0140-6736(19)33041-7

[31] Sanyal A.J. , Chalasani N., Kowdley K.V., McCullough A., Diehl A.M., Bass N.M., Neuschwander-Tetri B.A., Lavine J.E., Tonascia J., Unalp A. et al. Pioglitazone, vitamin e, or placebo for nonalcoholic steatohepatitis. N. Engl. J. Med. 2010; 362:1675–1685. 20427778 10.1056/NEJMoa0907929 PMC2928471

[32] Dewidar B. , Meyer C., Dooley S., Meindl-Beinker A.N. TGF-β in hepatic stellate cell activation and liver fibrogenesis-updated 2019. Cells. 2019; 8:1419. 31718044 10.3390/cells8111419 PMC6912224

[33] Nair B. , Nath L.R. Inevitable role of TGF-β1 in progression of nonalcoholic fatty liver disease. J. Recept. Signal Transduct. Res. 2020; 40:195–200. 32054379 10.1080/10799893.2020.1726952

[34] Fabregat I. , Moreno-Càceres J., Sánchez A., Dooley S., Dewidar B., Giannelli G., Ten Dijke P. TGF-β signalling and liver disease. FEBS J. 2016; 283:2219–2232. 26807763 10.1111/febs.13665

[35] Wang J. , Wu X., Jiang M., Tai G. Mechanism by which TRAF6 participates in the immune regulation of autoimmune diseases and cancer. Biomed. Res. Int. 2020; 2020:4607197. 33294443 10.1155/2020/4607197 PMC7714562

[36] Li J.J. , Luo J., Lu J.N., Liang X.N., Luo Y.H., Liu Y.R., Yang J., Ding H., Qin G.H., Yang L.H. et al. Relationship between TRAF6 and deterioration of HCC: an immunohistochemical and in vitro study. Cancer Cell Int. 2016; 16:76. 27708550 10.1186/s12935-016-0352-z PMC5041287

[37] Teratani T. , Tomita K., Suzuki T., Oshikawa T., Yokoyama H., Shimamura K., Tominaga S., Hiroi S., Irie R., Okada Y. et al. A high-cholesterol diet exacerbates liver fibrosis in mice via accumulation of free cholesterol in hepatic stellate cells. Gastroenterology. 2012; 142:152–164. 21995947 10.1053/j.gastro.2011.09.049

[38] Seki E. , De Minicis S., Osterreicher C.H., Kluwe J., Osawa Y., Brenner D.A., Schwabe R.F. TLR4 enhances TGF-beta signaling and hepatic fibrosis. Nat. Med. 2007; 13:1324–1332. 17952090 10.1038/nm1663

[39] Yahagi N. , Shimano H., Matsuzaka T., Sekiya M., Najima Y., Okazaki S., Okazaki H., Tamura Y., Iizuka Y., Inoue N. et al. p53 involvement in the pathogenesis of fatty liver disease. J. Biol. Chem. 2004; 279:20571–20575. 14985341 10.1074/jbc.M400884200

[40] Weng H. , Mertens P.R., Gressner A.M., Dooley S. IFN-gamma abrogates profibrogenic TGF-beta signaling in liver by targeting expression of inhibitory and receptor smads. J. Hepatol. 2007; 46:295–303. 17125875 10.1016/j.jhep.2006.09.014

[41] Holmes D. Liver: paradigm shift in the immunopathogenesis of NAFLD. Nat. Rev. Endocrinol. 2017; 13:500. 10.1038/nrendo.2017.95 28707681

[42] Seo W. , Eun H.S., Kim S.Y., Yi H.S., Lee Y.S., Park S.H., Jang M.J., Jo E., Kim S.C., Han Y.M. et al. Exosome-mediated activation of toll-like receptor 3 in stellate cells stimulates interleukin-17 production by γδ t cells in liver fibrosis. Hepatology. 2016; 64:616–631. 27178735 10.1002/hep.28644

[43] Xin Z. , Ma Z., Hu W., Jiang S., Yang Z., Li T., Chen F., Jia G., Yang Y. FOXO1/3: potential suppressors of fibrosis. Ageing Res. Rev. 2018; 41:42–52. 29138094 10.1016/j.arr.2017.11.002

[44] Miele L. , Beale G., Patman G., Nobili V., Leathart J., Grieco A., Abate M., Friedman S.L., Narla G., Bugianesi E. et al. The Kruppel-like factor 6 genotype is associated with fibrosis in nonalcoholic fatty liver disease. Gastroenterology. 2008; 135:282–291. 18515091 10.1053/j.gastro.2008.04.004 PMC2891245

[45] Ghiassi-Nejad Z. , Hernandez-Gea V., Woodrell C., Lang U.E., Dumic K., Kwong A., Friedman S.L. Reduced hepatic stellate cell expression of Kruppel-like factor 6 tumor suppressor isoforms amplifies fibrosis during acute and chronic rodent liver injury. Hepatology. 2013; 57:786–796. 22961688 10.1002/hep.26056 PMC3522757

[46] Yin L. , Liu M.X., Li W., Wang F.Y., Tang Y.H., Huang C.X. Over-Expression of inhibitor of differentiation 2 attenuates post-infarct cardiac fibrosis through inhibition of TGF-β1/Smad3/HIF-1α/IL-11 signaling pathway. Front. Pharmacol. 2019; 10:1349. 31803053 10.3389/fphar.2019.01349 PMC6876274

[47] Kaur D. , Sharma V., Deshmukh R. Activation of microglia and astrocytes: a roadway to neuroinflammation and alzheimer's disease. Inflammopharmacology. 2019; 27:663–677. 30874945 10.1007/s10787-019-00580-x

[48] Chen J.L. , Lu X.J., Zou K.L., Ye K. Krüppel-like factor 2 promotes liver steatosis through upregulation of CD36. J. Lipid Res. 2014; 55:32–40. 23861552 10.1194/jlr.M039453 PMC3927469

[49] Friedman S.L. Hepatic stellate cells: protean, multifunctional, and enigmatic cells of the liver. Physiol. Rev. 2008; 88:125–172. 18195085 10.1152/physrev.00013.2007 PMC2888531

[50] Glyn-Jones S. , Palmer A.J., Agricola R., Price A.J., Vincent T.L., Weinans H., Carr A.J. Osteoarthritis. Lancet. 2015; 386:376–387. 25748615 10.1016/S0140-6736(14)60802-3

[51] Nelson A.E. Osteoarthritis year in review 2017: clinical. Osteoarthritis Cartilage. 2018; 26:319–325. 29229563 10.1016/j.joca.2017.11.014 PMC5835411

[52] Choi M.C. , Jo J., Park J., Kang H.K., Park Y. NF-κB signaling pathways in osteoarthritic cartilage destruction. Cells. 2019; 8:734. 31319599 10.3390/cells8070734 PMC6678954

[53] Rigoglou S. , Papavassiliou A.G. The NF-κB signalling pathway in osteoarthritis. Int. J. Biochem. Cell Biol. 2013; 45:2580–2584. 24004831 10.1016/j.biocel.2013.08.018

[54] Chen D.H. , Zheng G., Zhong X.Y., Lin Z.H., Yang S.W., Liu H.X., Shang P. Oroxylin a attenuates osteoarthritis progression by dual inhibition of cell inflammation and hypertrophy. Food Funct. 2021; 12:328–339. 33300913 10.1039/d0fo02159h

[55] Pei W. , Huang X., Ni B., Zhang R., Niu G., You H. Selective STAT3 inhibitor alantolactone ameliorates osteoarthritis via regulating chondrocyte autophagy and cartilage homeostasis. Front. Pharmacol. 2021; 12:730312. 34650433 10.3389/fphar.2021.730312 PMC8505527

[56] He L. , Pan Y., Yu J., Wang B., Dai G., Ying X. Decursin alleviates the aggravation of osteoarthritis via inhibiting PI3K-Akt and NF-kB signal pathway. Int. Immunopharmacol. 2021; 97:107657. 33878544 10.1016/j.intimp.2021.107657

[57] Liu D.D. , Zhang B.L., Yang J.B., Zhou K. Celastrol ameliorates endoplasmic stress-mediated apoptosis of osteoarthritis via regulating ATF-6/CHOP signalling pathway. J. Pharm. Pharmacol. 2020; 72:826–835. 32201950 10.1111/jphp.13250

[58] Gratal P. , Lamuedra A., Mediero A., Herrero-Beaumont G., Largo R. The ginger derivate 6-shogaol as a treatment in osteoarthritis. Modulation of chondrocyte hypertrophy and matrix calcification. Osteoarthritis Cartilage. 2018; 26:S73–S74.

[59] Wang W. , Ha C., Lin T., Wang D., Wang Y., Gong M. Celastrol attenuates pain and cartilage damage via SDF-1/CXCR4 signalling pathway in osteoarthritis rats. J. Pharm. Pharmacol. 2018; 70:81–88. 28994112 10.1111/jphp.12835

[60] Sun K. , Luo J., Guo J., Yao X., Jing X., Guo F. The PI3K/AKT/mTOR signaling pathway in osteoarthritis: a narrative review. Osteoarthritis Cartilage. 2020; 28:400–409. 32081707 10.1016/j.joca.2020.02.027

[61] Pal B. , Endisha H., Zhang Y., Kapoor M. mTOR: a potential therapeutic target in osteoarthritis?. Drugs R. D. 2015; 15:27–36. 25688060 10.1007/s40268-015-0082-z PMC4359178

[62] Zhang Y. , Weng Q., Chen J., Li M., Han J. Oroxylin a attenuates IL-1β- induced inflammatory reaction via inhibiting the activation of the ERK and PI3K/AKT signaling pathways in osteoarthritis chondrocytes. Exp. Ther. Med. 2021; 21:388. 33680110 10.3892/etm.2021.9819 PMC7918508

[63] Katsara O. , Attur M., Ruoff R., Abramson S.B., Kolupaeva V. Increased activity of the chondrocyte translational apparatus accompanies osteoarthritic changes in human and rodent knee cartilage. Arthritis Rheumatol. 2017; 69:586–597. 27696794 10.1002/art.39947 PMC5329137

[64] Hamilton J.L. , Nagao M., Levine B.R., Chen D., Olsen B.R., Im H.J. Targeting VEGF and its receptors for the treatment of osteoarthritis and associated pain. J. Bone Miner. Res. 2016; 31:911–924. 27163679 10.1002/jbmr.2828 PMC4863467

[65] Liu Z. , Wang H., Wang S., Gao J., Niu L. PARP-1 inhibition attenuates the inflammatory response in the cartilage of a rat model of osteoarthritis. Bone Joint Res. 2021; 10:401–410. 34254815 10.1302/2046-3758.107.BJR-2020-0200.R2 PMC8333032

