# Supplementary material for: ChemPert: mapping between chemical perturbation and transcriptional response for non-cancer cells
Source: Nucleic Acids Res. 2022 Oct 6;51(D1):D877–89. doi: 10.1093/nar/gkac862 (PMC9825489; doi:10.1093/nar/gkac862)
Supplement: gkac862_Supplemental_Files [file gkac862_supplemental_files.zip › Supplementary_information_revised2.pdf]

Supplementary captions

Supplementary Table S1
GSEA for TFs commonly up-regulated in both NASH diet models and disease states after pioglitazone perturbation.

Supplementary Table S2
GSEA for TFs commonly down-regulated in both NASH diet models and disease states after pioglitazone perturbation.

Supplementary Table S3
GSEA for TFs commonly up-regulated in both NASH diet models and disease states after vitamine E perturbation.

Supplementary Table S4
Predicted perturbagens and signalling proteins for the treatment of OA.

Supplementary figures

A

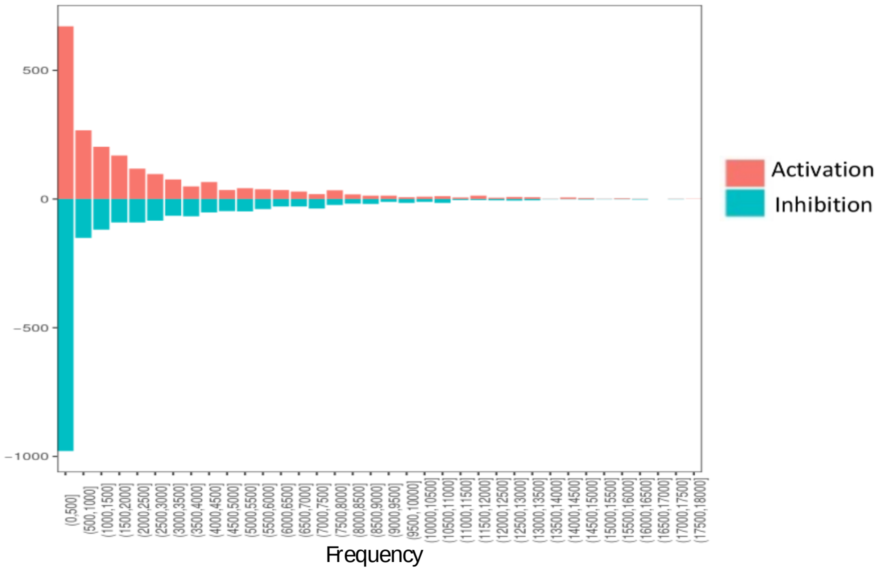
500
0
−500
−1000
Frequency
Activation
Inhibition

B

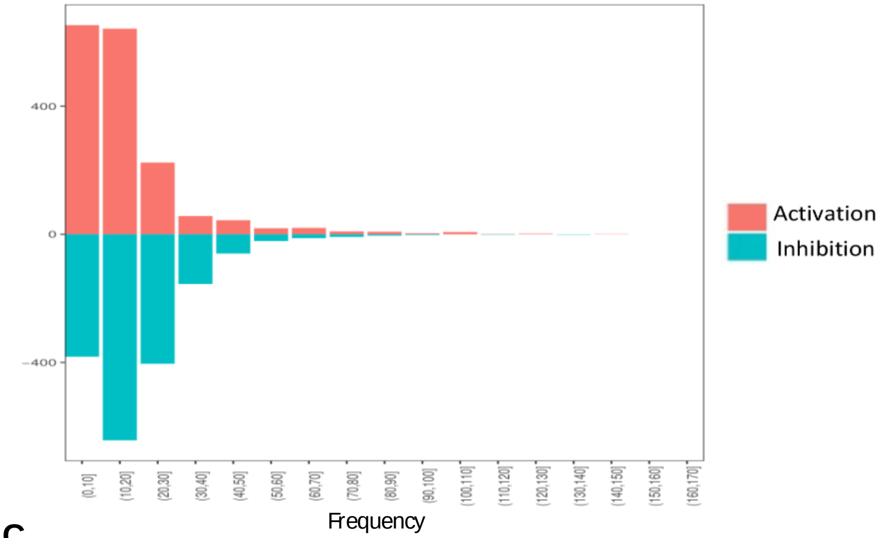
400
0
−400
Frequency
Activation
Inhibition

C

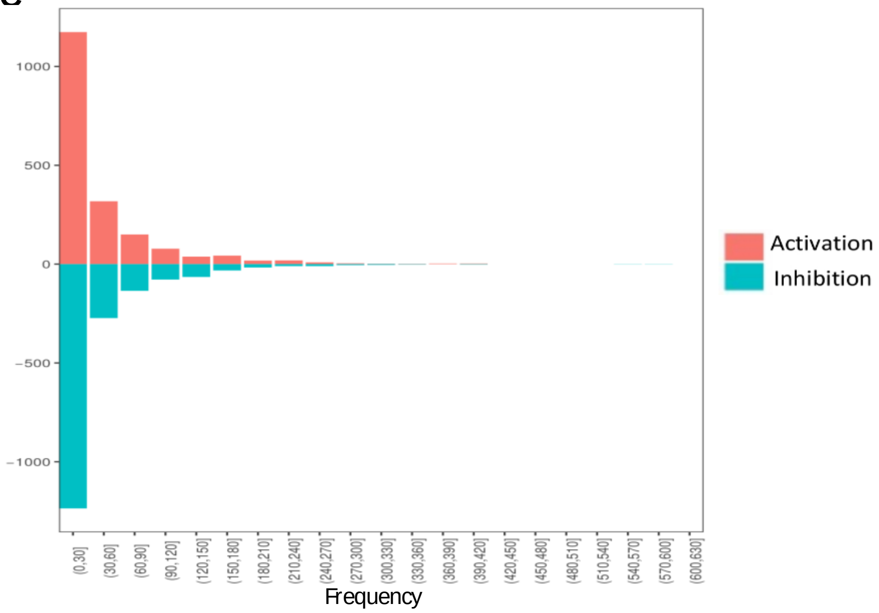
1000
500
0
−500
−1000
Frequency
Activation
Inhibition

Figure S1. Distribution of perturbagen frequency by species in the ChemPert database. (**A**) Human. (**B**) Mouse. (**C**) Rat. X-axis represents the frequency of perturbagens, and y-axis represents the number of perturbagens with corresponding frequency.

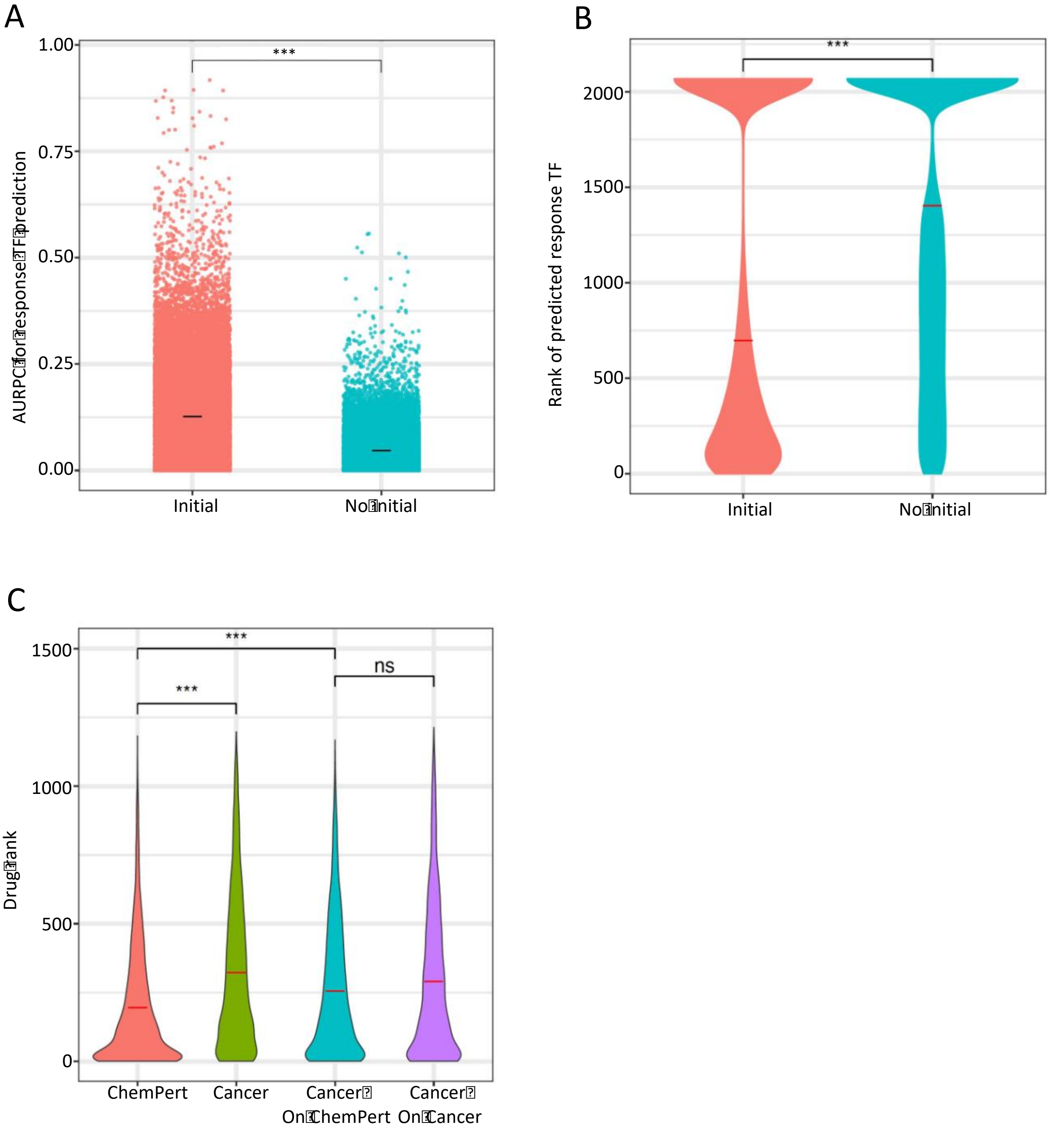

Figure S2. The evaluation of ChemPert database. (**A**) AURPC for response TF prediction with and without gene expression of initial cellular states. (**B**) Rank of predicted TF hits with and without gene expression of initial cellular states. Unpredicted TF targets were given the 2067th rank (number of considered TFs) (**C**) Rank of true perturbagens for benchmarking datasets predicted for non-cancer datasets using ChemPert database (ChemPert) and cancer database (Cancer), and predicted for cancer datasets using ChemPert database (Cancer on ChemPert) and cancer database (Cancer on Cancer).*** indicates statistical significance ($p < 0.001$) based on one-sided Wilcoxon rank-sum test. ns: not significant.

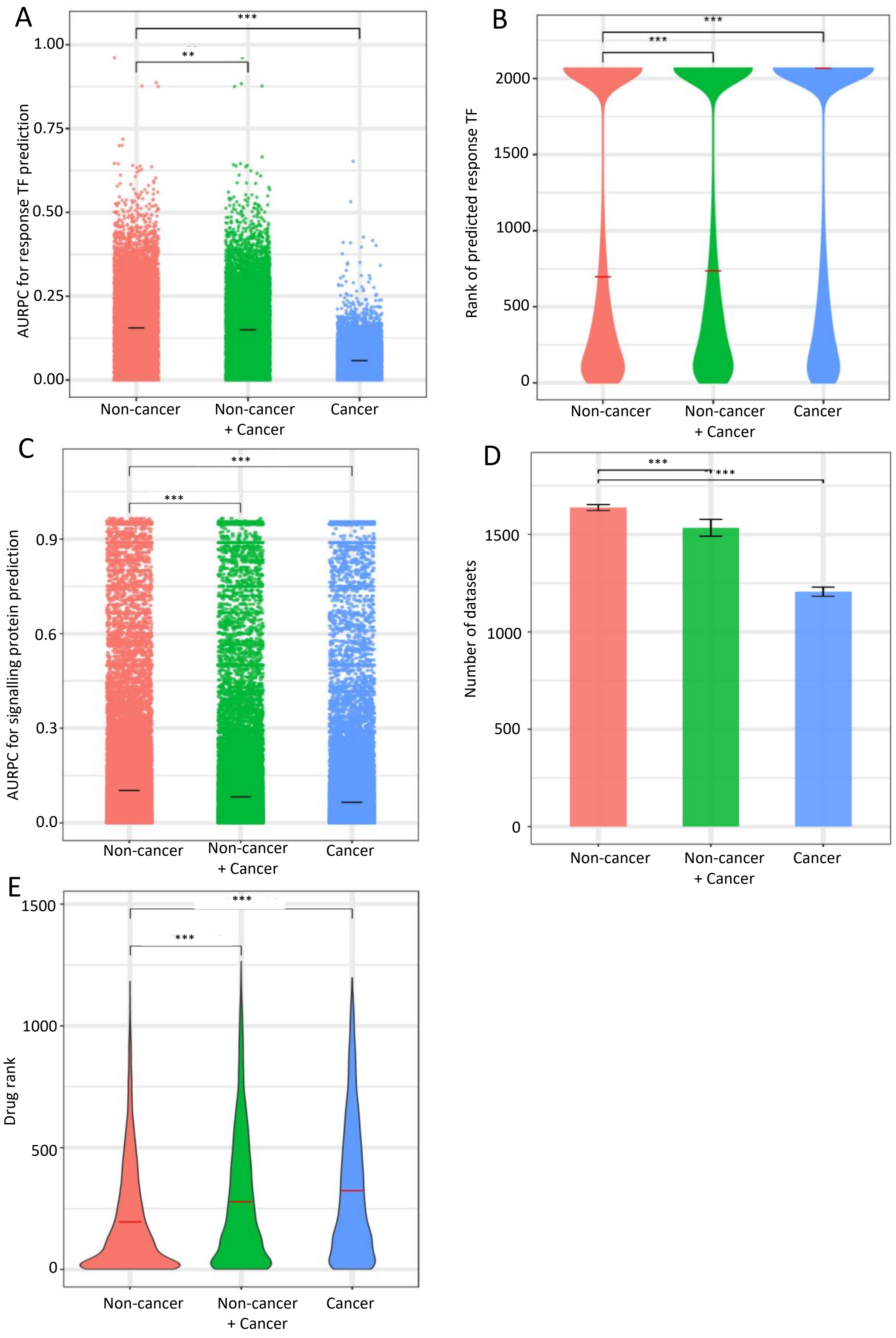
A
AURPC for response TF prediction
1.00
0.75
0.50
0.25
0.00
***
**
Non-cancer
Non-cancer
+ Cancer
Cancer
B
Rank of predicted response TF
2000
1500
1000
500
0
***
***
Non-cancer
Non-cancer
+ Cancer
Cancer
C
AURPC for signalling protein prediction
0.9
0.6
0.3
0.0
***
***
Non-cancer
Non-cancer
+ Cancer
Cancer
D
Number of datasets
1500
1000
500
0
***
***
Non-cancer
Non-cancer
+ Cancer
Cancer
E
Drug rank
1500
1000
500
0
***
***
Non-cancer
Non-cancer
+ Cancer
Cancer

Figure S3. Benchmarking with non-cancer and cancer combined database. (**A**) AURPC for response TF prediction based on three databases, Non-cancer (default ChemPert), non-cancer+cancer (both databases combine), Cancer (only cancer database). (**B**) Rank of predicted TF hits based on the three databases. Unpredicted TF targets were given the 2067th rank (number of considered TFs). (**C**) AURPC for signalling protein prediction. (**D**) Number of datasets with correct perturbagen prediction. (**E**) Predicted rank of true perturbagens. ** and *** indicate different levels of statistical significance ($p < 0.01$, $p < 0.001$, respectively) based on Wilcoxon rank-sum test.

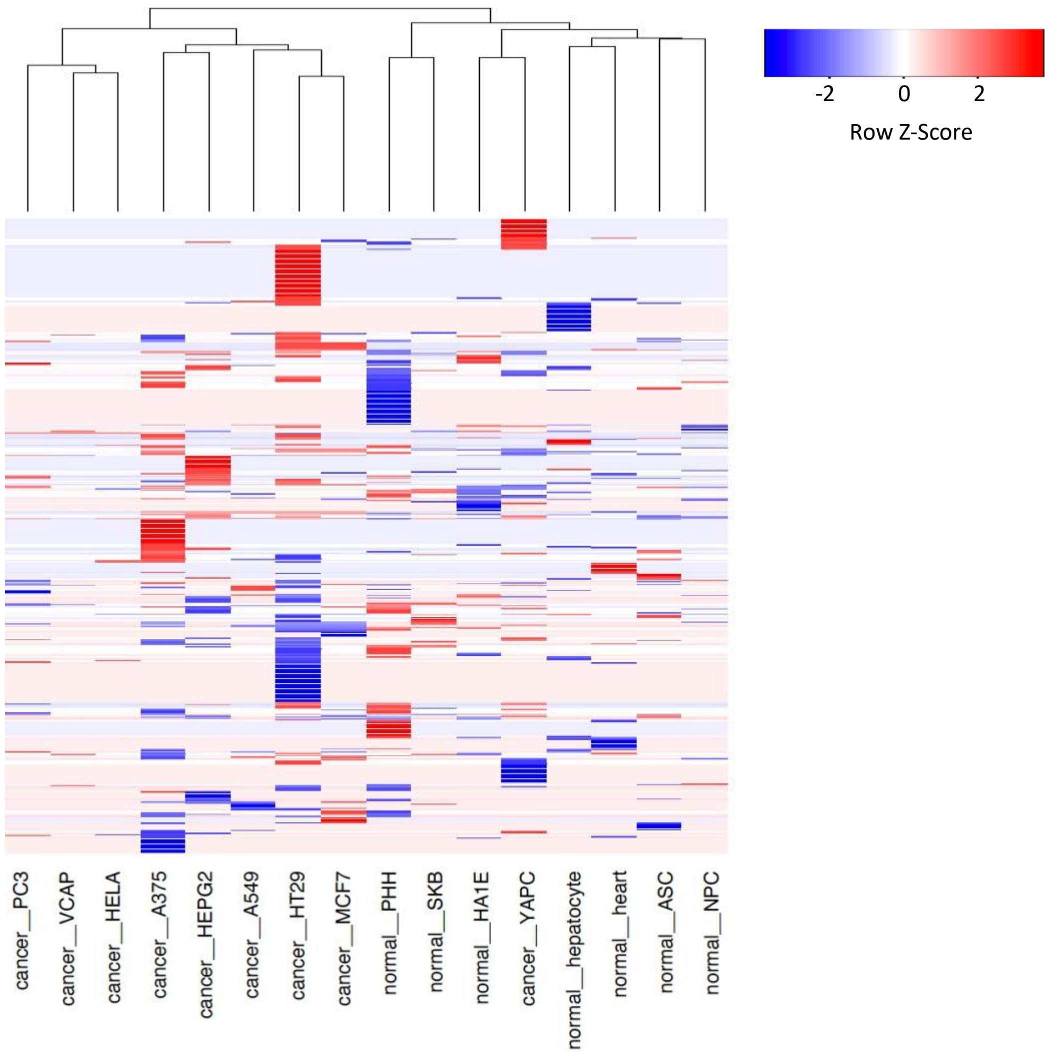

Figure S4. Hierarchical clustering of non-cancer and cancer response profiles to ranitidine. Clustering was performed using normalized log2 gene expression fold changes before and after perturbation. “Normal” indicates non-cancer cell types and “cancer” indicates cancer cell types.

A

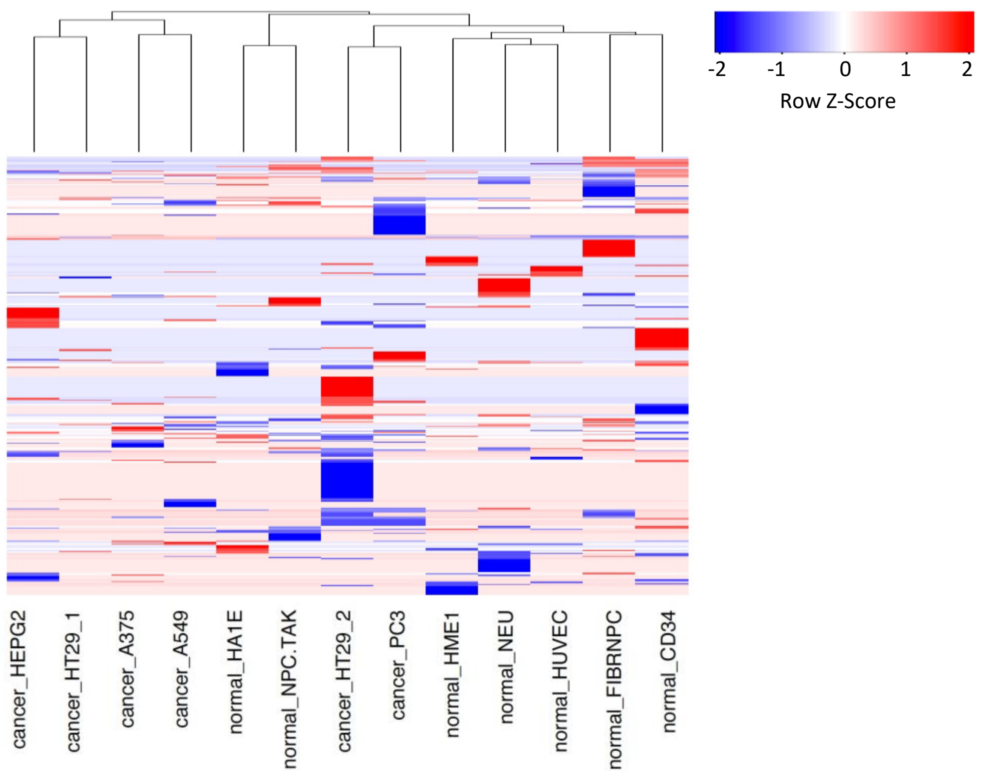
-2
-1
0
1
2
Row Z-Score
cancer_HEPG2
cancer_HT29_1
cancer_A375
cancer_A549
normal_HA1E
normal_NPC.TAK
cancer_HT29_2
cancer_PC3
normal_HME1
normal_NEU
normal_HUVEC
normal_FIBRNPC
normal_CD34

B

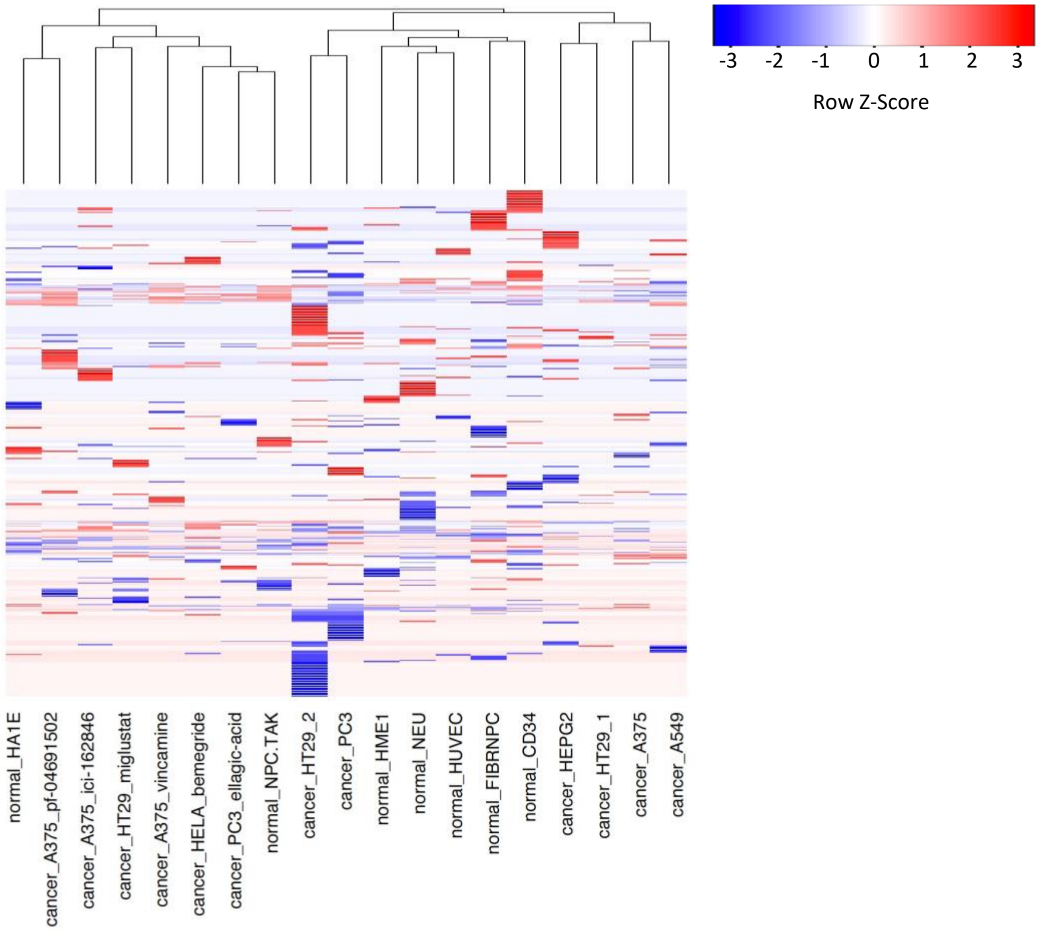
-3
-2
-1
0
1
2
3
Row Z-Score
normal_HA1E
cancer_A375_pf-04691502
cancer_A375_ici-162846
cancer_HT29_miglustat
cancer_A375_vincamine
cancer_HELA_bemegride
cancer_PC3_ellagic-acid
normal_NPC.TAK
cancer_HT29_2
cancer_PC3
normal_HME1
normal_NEU
normal_HUVEC
normal_FIBRNPC
normal_CD34
cancer_HEPG2
cancer_HT29_1
cancer_A375
cancer_A549

Figure S5. Hierarchical clustering of non-cancer and cancer response profiles. (**A)** Tranylcyorimine perturbation. (**B**) Tranylcyorimine perturbed non-cancer cells and cancer cells with other perturbations. Clustering was performed using normalized log2 gene expression fold changes before and after perturbation. “Normal” indicates non-cancer cell types and “cancer” indicates cancer cell types.

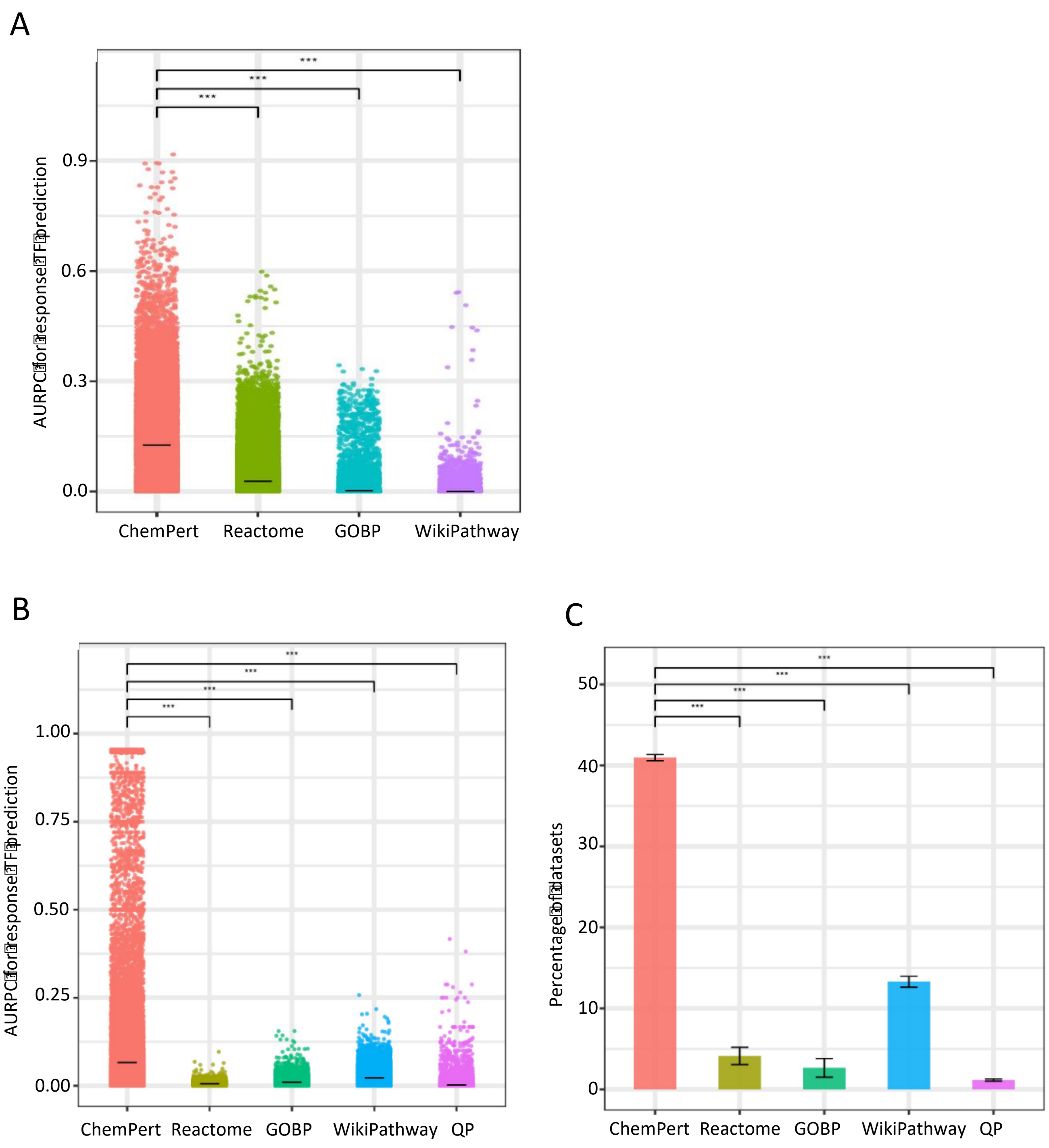

Figure S6. Benchmarking with GSEA-based signalling pathway inference approaches. (**A**) AURPC for response TF prediction. (**B**) AURPC for response signalling protein target prediction. (**C**) Percentage of datasets where correct perturbagens were predicted. *** indicates statistical significance ($p < 0.001$) based on Wilcoxon rank-sum test. QP: QuatenaryProd.

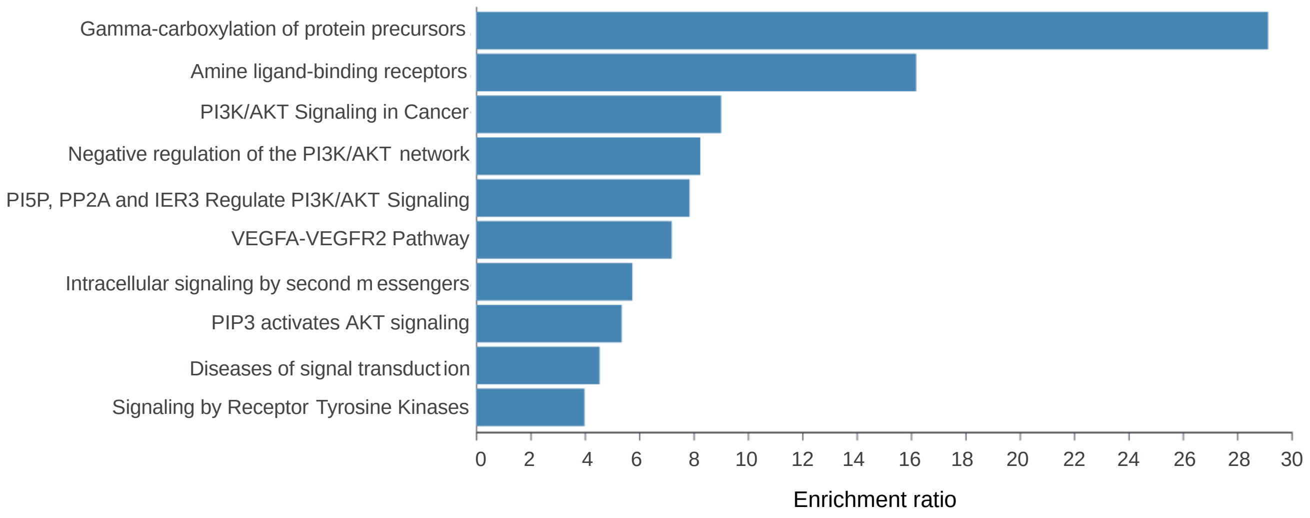

Figure S7. The over representation analysis of predicted signalling proteins for the treatment of OA.

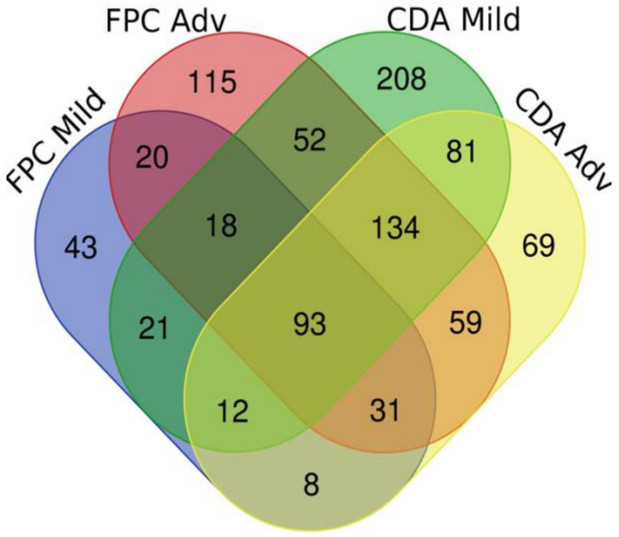

Figure S8. Venn diagram showing overlaps of predicted perturbagens among different diets and disease states of NASH models.

Supplementary Note

Description of each TF predicted after OCA perturbation.

ATF6 has been identified as an inhibitor of the transcription of sterol-regulatory element-binding protein (*Srebp*), a master regulator of fatty acid synthesis (1,2). Furthermore, upregulation of this TF has also been shown to improve glucose tolerance and insulin resistance (IR), features closely related to NAFLD (3). **HBP1** is also an inhibitor of *Srebp* (4); while **BTG1** inhibits the expression of stearoyl-CoA desaturase 1 (*Scd1*), another enzyme also involved in the synthesis of fatty acids (5). **SAP18** is known to decrease hepatic lipid levels by increasing microsomal TG transfer protein (MTTP) expression levels, required for the assembly and secretion of lipoproteins (6). Regarding the improvement of steatohepatitis, both **PPAR-D** and **PPARG** play a regulatory role in lipid metabolism but also in inflammation (7). PPARD is largely expressed in tissues with high mitochondrial and peroxisomal β-oxidative activity, as it controls processes related to fatty acid metabolism and inflammation (8). There is some controversy about the role of PPARG in NAFLD pathology, as its overexpression is a general property of steatotic livers, but it has been proofed that its activation reduces hepatic steatosis (9). PPARG stimulation lessens free fatty acid (FFA) levels (through adipogenesis), attenuates hepatic glucose production, and improves insulin sensitivity (10). Furthermore, it inhibits the expression of inflammatory cytokines and direct the differentiation of immune cells towards anti-inflammatory phenotypes (11). Another characteristic of NAFLD progression is hepatocyte lipoapoptosis, which plays a key role in mediating hepatic inflammation and correlates with the stage of fibrosis. Elevated concentrations of FFA induce hepatocyte death, through induction of inhibitor of apoptosis protein (cIAP1 or **BIRC2**) degradation (12). Forkhead box proteins O1 (**FOXO1**) is a potent inhibitor of fibroblast activation and subsequent extracellular matrix (ECM) production and can ameliorate fibrosis levels in numerous organs, including the liver; being reported as a promising target for anti-fibrosis therapy (13). There is a close association between NASH and IR (14), and even it has been hypothesized that genetic polymorphisms related to insulin resistance play a role in NAFLD susceptibility (15). In accordance, it is described that hyperinsulinemia inactivates FOXO1 in HSCs, resulting in HSC activation, which may result in fibrosis development in NASH (16). In line, elevated insulin levels in fibrosis have shown to enhance NK cell cytotoxic activity toward activated HSCs, thereby preventing fibrosis progression (17). Therefore, an increase in insulin receptor (**INSR**) levels corroborates the anti-fibrotic effect of this drug. In addition, **KLF6** expression has been reported to be increased in association with increased steatosis, inflammation and fibrosis in NAFLD livers (18). However, its overexpression is known to abrogate the up-regulation of both αSMA and collagen 1 (19). It is therefore an anti-fibrotic factor that is already induced during the disease to counteract liver damage. Therefore, the induction of an even higher expression would improve the pathology. In fact, two mechanisms of action have been described: a direct transcriptional repression of target fibrogenic genes and an increased apoptosis of activated HSCs (20). **DACH1** expression is lower in the fatty liver and cirrhosis, but also in liver cancer tissues, with a negative correlation with pathologic grade, so it may play a protective role in liver diseases (21). **RYBP**, a member of the polycomb group (PcG) proteins, has been described to be downregulated in hepatocellular carcinoma (HCC) tissue samples, and its low expression has an independent predictor of a poor prognosis in patients with HCC (22). Thus, reactivating RYBP in cancer cells may provide an effective and

safe therapeutic approach to HCC therapy (23,24). Finally, **ZFP36L1** suppresses epithelial mesenchymal transition, suggesting an important role in liver fibrosis and the development of HCC (25).

Regarding the downregulated TFs, **CNOT3** induces increased metabolic rates in hepatic and adipose tissues and enhances glucose tolerance (26); **CREB3L3** is involved in fatty acid oxidation (27); **REPIN1** is related to lipid and glucose metabolism and obesity regulation (28); and **STAT1** belongs to the STAT family with inflammatory biological functions that have been associated with NAFLD and NASH (29,30). **CCNE1** is indispensable for the activation, proliferation, and survival of HSCs and thus promotes the synthesis of ECM and liver fibrogenesis (31,32). **ETS1** is elevated in a NASH preclinical model and its knockdown alleviated hepatocyte apoptosis, inflammation, and fibrosis. (30,33). **HDAC6** and **HDAC9** are widely involved in the pathogenesis of NAFLD and have been postulated as potential to become therapeutic targets (22,34). Expression of **HLF** has been detected in fibrotic livers and plays a role during HSC activation, while loss of HLF impairs HSC activation and attenuates liver fibrosis (35). Both **PLAGL1** and **SOX4**, have been identified to be upregulated in activated HSCs as well as in fibrotic liver tissues (36). **TRIM16** and **TRIM29** are involved in the regulation of fibrosis-related factors and signaling pathways (37). In relation to disease progression, downregulation of TFs involved in the development of HCC was predicted: B-cell leukemia 3 (**BCL3**) has been identified to induce the activation of macrophages during hepatocarcinogenesis, thereby promoting the occurrence of HCC (38); **MYCBP** was verified in human HCC tissues, patients that poorly expressed MYCBP had significantly longer disease-free survival and overall survival (39); and hepatic **SMARCA4** predicts HCC recurrence and promotes tumour cell proliferation (40).

Description of each perturbagens predicted for four NASH mouse models

Currently no drug has been approved as a treatment for NASH. Nevertheless, following this comment by the referee, we first identified differentially expressed TFs between the control vs 1) FPC_Mild, 2) FPC_Adv, 3) CDA_Mild, and 4) CDA_Adv mouse transcriptomics data from (*41*) and then applied our perturbagen prediction tool to each of these DETF sets. The result (Figure S6) shows 93 common predicted perturbagens across all the four conditions. As expected, none of them are known to cure the disease, however, some of them have been implicated for partial treatments. For example, chalcone has been reported for its protective effects on NASH liver in preclinical study (42, 43). Administration of fucoxanthin to mice has been shown to prevent early phase of NASH by inhibiting steatosis, inflammation and fibrosis (44, 45). Similarly, supplementation of honokiol has been able to attenuate NASH in mice possibly by macrophage polarization and glucocorticoid receptor translocation (46, 47). Indeed, glucocorticoid and glucocorticoid receptor agonist were also among the 96 common predicted perturbagens. A phenolic compound, pterostilbene, has also been demonstrated to partially prevent NASH progression by reducing oxidative stress and steatohepatitis (48, 49). Moreover, a hedgehog signaling pathway inhibitor, vismodegib, has been reported to reduce steatohepatitis in mice (50, 51).

The 59 predicted perturbagens common to the two advanced NASH models included VEGF, VEGFA, FGFs and fiin-1, which are well-known for their roles in fibrosis attenuation (52, 53). Hydroxytyrosol has been shown to prevent progression of NASH to irreversible states (54, 55). Acarbose, an α-glucosidase inhibitor, has been reported to reduce steatosis and the risk of NASH progression to

advanced stages (56-58). The prevention of steatosis progression has also been implicated for caffeic acid (59-61). Cyclopamine, another hedgehog signaling pathway inhibitor, has been known for its effect on reducing fibrosis (62-64). Inhibition of sphingosine signalling has been reported to prevent NASH progression of steatohepatitis and to hepatocarcinoma (65-67). Finally, a selective inhibitor of ACSL4, abemaciclib, has been implicated in reducing NASH symptoms (68).

Finally, pioglitazone was predicted for the CDA Mild condition and vitamin E was predicted for CDA Mild and FPC Adv conditions. This result suggests that these compounds could be effective for some NASH states but may not be a universal cure on their own.

Taken together, the perturbagen prediction tool was able to identify several compounds reported to be able to partially treat mouse NASH models and predict novel ones for experimental follow-ups. Since these compounds seem to target different aspects of NASH, combinatorial therapies are likely needed for effective cures.

Reference

1. Han, J., Murthy, R., Wood, B., Song, B., Wang, S., Sun, B., Malhi, H. and Kaufman, R.J. (2013) ER stress signalling through eIF2α and CHOP, but not IRE1α, attenuates adipogenesis in mice. *Diabetologia*, **56**, 911-924.
2. Zeng, L., Lu, M., Mori, K., Luo, S., Lee, A.S., Zhu, Y. and Shyy, J.Y. (2004) ATF6 modulates SREBP2-mediated lipogenesis. *Embo j*, **23**, 950-958.
3. Sun, X., Li, W., Deng, Y., Dong, B., Sun, Y., Xue, Y. and Wang, Y. (2018) Hepatic conditional knockout of ATF6 exacerbates liver metabolic damage by repressing autophage through MTOR pathway. *Biochem Biophys Res Commun*, **505**, 45-50.
4. Wu, H., Ng, R., Chen, X., Steer, C.J. and Song, G. (2016) MicroRNA-21 is a potential link between non-alcoholic fatty liver disease and hepatocellular carcinoma via modulation of the HBP1-p53-Srebp1c pathway. *Gut*, **65**, 1850-1860.
5. Xiao, F., Deng, J., Guo, Y., Niu, Y., Yuan, F., Yu, J., Chen, S. and Guo, F. (2016) BTG1 ameliorates liver steatosis by decreasing stearoyl-CoA desaturase 1 (SCD1) abundance and altering hepatic lipid metabolism. *Sci Signal*, **9**, ra50.
6. Makishima, S., Boonvisut, S., Ishizuka, Y., Watanabe, K., Nakayama, K. and Iwamoto, S. (2015) Sin3A-associated protein, 18 kDa, a novel binding partner of TRIB1, regulates MTTP expression. *J Lipid Res*, **56**, 1145-1152.
7. Boeckmans, J., Natale, A., Rombaut, M., Buyl, K., Rogiers, V., De Kock, J., Vanhaecke, T. and R, M.R. (2019) Anti-NASH Drug Development Hitches a Lift on PPAR Agonism. *Cells*, **9**.
8. Palomer, X., Barroso, E., Pizarro-Delgado, J., Peña, L., Botteri, G., Zarei, M., Aguilar, D., Montori-Grau, M. and Vázquez-Carrera, M. (2018) PPARβ/δ: A Key Therapeutic Target in Metabolic Disorders. *Int J Mol Sci*, **19**.

9. Morán-Salvador, E., López-Parra, M., García-Alonso, V., Titos, E., Martínez-Clemente, M., González-Périz, A., López-Vicario, C., Barak, Y., Arroyo, V. and Clària, J. (2011) Role for PPAR γ in obesity-induced hepatic steatosis as determined by hepatocyte- and macrophage-specific conditional knockouts. *Faseb j*, **25**, 2538-2550.
10. Pais, R., Moraru, I. and Ratziu, V. (2011) Glitazones for human nonalcoholic steatohepatitis. *Therap Adv Gastroenterol*, **4**, 325-334.
11. Martin, H. (2010) Role of PPAR-gamma in inflammation. Prospects for therapeutic intervention by food components. *Mutat Res*, **690**, 57-63.
12. Akazawa, Y. and Nakao, K. (2018) To die or not to die: death signaling in nonalcoholic fatty liver disease. *J Gastroenterol*, **53**, 893-906.
13. Xin, Z., Ma, Z., Hu, W., Jiang, S., Yang, Z., Li, T., Chen, F., Jia, G. and Yang, Y. (2018) FOXO1/3: Potential suppressors of fibrosis. *Ageing Res Rev*, **41**, 42-52.
14. Chitturi, S., Abeygunasekera, S., Farrell, G.C., Holmes-Walker, J., Hui, J.M., Fung, C., Karim, R., Lin, R., Samarasinghe, D., Liddle, C. *et al.* (2002) NASH and insulin resistance: Insulin hypersecretion and specific association with the insulin resistance syndrome. *Hepatology*, **35**, 373-379.
15. Nobakht, H., Mahmoudi, T., Sabzikarian, M., Tabaeian, S.P., Rezamand, G., Asadi, A., Farahani, H., Dabiri, R., Mansour-Ghanaei, F., Maleki, I. *et al.* (2020) INSULIN AND INSULIN RECEPTOR GENE POLYMORPHISMS AND SUSCEPTIBILITY TO NONALCOHOLIC FATTY LIVER DISEASE. *Arq Gastroenterol*, **57**, 203-208.
16. Adachi, M., Osawa, Y., Uchinami, H., Kitamura, T., Accili, D. and Brenner, D.A. (2007) The forkhead transcription factor FoxO1 regulates proliferation and transdifferentiation of hepatic stellate cells. *Gastroenterology*, **132**, 1434-1446.
17. Amer, J., Salhab, A., Noureddin, M., Doron, S., Abu-Tair, L., Ghantous, R., Mahamid, M. and Safadi, R. (2018) Insulin signaling as a potential natural killer cell checkpoint in fatty liver disease. *Hepatol Commun*, **2**, 285-298.
18. Wang, X.M. and Chen, D.F. (2007) [Effects of rosiglitazone on Kruppul-like factor 6 (KLF6) signaling in the livers of rats with nonalcoholic fatty liver fibrosis]. *Zhonghua Gan Zang Bing Za Zhi*, **15**, 649-653.
19. Miele, L., Beale, G., Patman, G., Nobili, V., Leathart, J., Grieco, A., Abate, M., Friedman, S.L., Narla, G., Bugianesi, E. *et al.* (2008) The Kruppel-like factor 6 genotype is associated with fibrosis in nonalcoholic fatty liver disease. *Gastroenterology*, **135**, 282-291.e281.
20. Ghiassi-Nejad, Z., Hernandez-Gea, V., Woodrell, C., Lang, U.E., Dumic, K., Kwong, A. and Friedman, S.L. (2013) Reduced hepatic stellate cell expression of Kruppel-like factor 6 tumor suppressor isoforms amplifies fibrosis during acute and chronic rodent liver injury. *Hepatology*, **57**, 786-796.
21. Yin, T. and Si, Z. (2014) Expression Analyses of the Key Members of Rdgn in the

Tissues of Benign Hepatic Diseases and Human Liver Cancers. *Annals of Oncology*, **25**, iv248.
22. Claveria-Cabello, A., Colyn, L., Arechederra, M., Urman, J.M., Berasain, C., Avila, M.A. and Fernandez-Barrena, M.G. (2020) Epigenetics in Liver Fibrosis: Could HDACs be a Therapeutic Target? *Cells*, **9**.
23. Wang, W., Cheng, J., Qin, J.J., Voruganti, S., Nag, S., Fan, J., Gao, Q. and Zhang, R. (2014) RYBP expression is associated with better survival of patients with hepatocellular carcinoma (HCC) and responsiveness to chemotherapy of HCC cells in vitro and in vivo. *Oncotarget*, **5**, 11604-11619.
24. Zhao, Q., Cai, W., Zhang, X., Tian, S., Zhang, J., Li, H., Hou, C., Ma, X., Chen, H., Huang, B. *et al.* (2017) RYBP Expression Is Regulated by KLF4 and Sp1 and Is Related to Hepatocellular Carcinoma Prognosis. *J Biol Chem*, **292**, 2143-2158.
25. Tarling, E.J., Clifford, B.L., Cheng, J., Morand, P., Cheng, A., Lester, E., Sallam, T., Turner, M. and de Aguiar Vallim, T.Q. (2017) RNA-binding protein ZFP36L1 maintains posttranscriptional regulation of bile acid metabolism. *J Clin Invest*, **127**, 3741-3754.
26. Morita, M., Oike, Y., Nagashima, T., Kadomatsu, T., Tabata, M., Suzuki, T., Nakamura, T., Yoshida, N., Okada, M. and Yamamoto, T. (2011) Obesity resistance and increased hepatic expression of catabolism-related mRNAs in Cnot3+/- mice. *Embo j*, **30**, 4678-4691.
27. Nakagawa, Y., Satoh, A., Tezuka, H., Han, S.I., Takei, K., Iwasaki, H., Yatoh, S., Yahagi, N., Suzuki, H., Iwasaki, Y. *et al.* (2016) CREB3L3 controls fatty acid oxidation and ketogenesis in synergy with PPARα. *Sci Rep*, **6**, 39182.
28. Abshagen, K., Mense, L., Fischer, F., Liebig, M., Schaeper, U., Navarro, G., Glass, Ä., Frank, M., Klöting, N. and Vollmar, B. (2019) Repin1 deficiency in liver tissue alleviates NAFLD progression in mice. *J Adv Res*, **16**, 99-111.
29. Grohmann, M., Wiede, F., Dodd, G.T., Gurzov, E.N., Ooi, G.J., Butt, T., Rasmiena, A.A., Kaur, S., Gulati, T., Goh, P.K. *et al.* (2018) Obesity Drives STAT-1-Dependent NASH and STAT-3-Dependent HCC. *Cell*, **175**, 1289-1306.e1220.
30. Steensels, S., Qiao, J. and Ersoy, B.A. (2020) Transcriptional Regulation in Non-Alcoholic Fatty Liver Disease. *Metabolites*, **10**.
31. Bangen, J.M., Hammerich, L., Sonntag, R., Baues, M., Haas, U., Lambertz, D., Longerich, T., Lammers, T., Tacke, F., Trautwein, C. *et al.* (2017) Targeting CCl(4) - induced liver fibrosis by RNA interference-mediated inhibition of cyclin E1 in mice. *Hepatology*, **66**, 1242-1257.
32. Nevzorova, Y.A., Bangen, J.M., Hu, W., Haas, U., Weiskirchen, R., Gassler, N., Huss, S., Tacke, F., Sicinski, P., Trautwein, C. *et al.* (2012) Cyclin E1 controls proliferation of hepatic stellate cells and is essential for liver fibrogenesis in mice. *Hepatology*, **56**, 1140-1149.

33. Liu, D., Wang, K., Li, K., Xu, R., Chang, X., Zhu, Y., Sun, P. and Han, X. (2019) Ets-1 deficiency alleviates nonalcoholic steatohepatitis via weakening TGF-β1 signaling-mediated hepatocyte apoptosis. *Cell Death Dis*, **10**, 458.
34. Fu, S., Yu, M., Tan, Y. and Liu, D. (2021) Role of histone deacetylase on nonalcoholic fatty liver disease. *Expert Rev Gastroenterol Hepatol*, **15**, 353-361.
35. Xiang, D.M., Sun, W., Ning, B.F., Zhou, T.F., Li, X.F., Zhong, W., Cheng, Z., Xia, M.Y., Wang, X., Deng, X. *et al.* (2018) The HLF/IL-6/STAT3 feedforward circuit drives hepatic stellate cell activation to promote liver fibrosis. *Gut*, **67**, 1704-1715.
36. He, L., Yuan, H., Liang, J., Hong, J. and Qu, C. (2020) Expression of hepatic stellate cell activation-related genes in HBV-, HCV-, and nonalcoholic fatty liver disease-associated fibrosis. *PLoS One*, **15**, e0233702.
37. Qian, H. and Chen, L. (2021) TRIM proteins in fibrosis. *Biomed Pharmacother*, **144**, 112340.
38. Huang, Y., Yang, X., Meng, Y., Shao, C., Liao, J., Li, F., Li, R., Jing, Y. and Huang, A. (2021) The hepatic senescence-associated secretory phenotype promotes hepatocarcinogenesis through Bcl3-dependent activation of macrophages. *Cell Biosci*, **11**, 173.
39. Zhu, X.X., Li, J.H., Cai, J.P., Hou, X., Huang, C.S., Huang, X.T., Wang, J.Q., Li, S.J., Xu, Q.C. and Yin, X.Y. (2019) EYA4 inhibits hepatocellular carcinoma by repressing MYCBP by dephosphorylating β-catenin at Ser552. *Cancer Sci*, **110**, 3110-3121.
40. Chen, Z., Lu, X., Jia, D., Jing, Y., Chen, D., Wang, Q., Zhao, F., Li, J., Yao, M., Cong, W. *et al.* (2018) Hepatic SMARCA4 predicts HCC recurrence and promotes tumour cell proliferation by regulating SMAD6 expression. *Cell Death Dis*, **9**, 59.
41. Loft, A., Alfaro, A.J., Schmidt, S.F., Pedersen, F.B., Terkelsen, M.K., Puglia, M., Chow, K.K., Feuchtinger, A., Troullinaki, M., Maida, A. et al. (2021) Liver-fibrosis-activated transcriptional networks govern hepatocyte reprogramming and intra-hepatic communication. Cell Metab, 33, 1685-1700.e1689.
42. Karimi-Sales E, Mohaddes G, Alipour MR. Chalcones as putative hepatoprotective agents: Preclinical evidence and molecular mechanisms. Pharmacol Res. 2018 Mar;129:177-187. doi: 10.1016/j.phrs.2017.11.022. Epub 2017 Nov 23. PMID: 29175112.
43. Karimi-Sales E, Ebrahimi-Kalan A, Alipour MR. Preventive effect of trans-chalcone on non-alcoholic steatohepatitis: Improvement of hepatic lipid metabolism. Biomed Pharmacother. 2019 Jan;109:1306-1312. doi: 10.1016/j.biopha.2018.10.196. Epub 2018 Nov 9. PMID: 30551380.
44. Shih PH, Shiue SJ, Chen CN, Cheng SW, Lin HY, Wu LW, Wu MS. Fucoidan and Fucoxanthin Attenuate Hepatic Steatosis and Inflammation of NAFLD through Modulation of Leptin/Adiponectin Axis. Mar Drugs. 2021 Mar 12;19(3):148. doi: 10.3390/md19030148. PMID: 33809062; PMCID: PMC8001566.

45. Takatani N, Kono Y, Beppu F, Okamatsu-Ogura Y, Yamano Y, Miyashita K, Hosokawa M. Fucoxanthin inhibits hepatic oxidative stress, inflammation, and fibrosis in diet-induced nonalcoholic steatohepatitis model mice. Biochem Biophys Res Commun. 2020 Jul 23;528(2):305-310. doi: 10.1016/j.bbrc.2020.05.050. Epub 2020 May 29. PMID: 32475638.
46. Zhong X, Liu H. Honokiol attenuates diet-induced non-alcoholic steatohepatitis by regulating macrophage polarization through activating peroxisome proliferator-activated receptor γ. J Gastroenterol Hepatol. 2018 Feb;33(2):524-532. doi: 10.1111/jgh.13853. PMID: 28670854.
47. Okuda K, Umemura A, Umemura S, Kataoka S, Taketani H, Seko Y, Nishikawa T, Yamaguchi K, Moriguchi M, Kanbara Y, Arbiser JL, Shima T, Okanoue T, Karin M, Itoh Y. Honokiol Prevents Non-Alcoholic Steatohepatitis-Induced Liver Cancer via EGFR Degradation through the Glucocorticoid Receptor-MIG6 Axis. Cancers (Basel). 2021 Mar 25;13(7):1515. doi: 10.3390/cancers13071515. PMID: 33806040; PMCID: PMC8037653.
48. Gómez-Zorita S, Milton-Laskíbar I, Aguirre L, Fernández-Quintela A, Xiao J, Portillo MP. Effects of Pterostilbene on Diabetes, Liver Steatosis and Serum Lipids. Curr Med Chem. 2021;28(2):238-252. doi: 10.2174/0929867326666191029112626. PMID: 31663469.
49. Gómez-Zorita S, González-Arceo M, Trepiana J, Aguirre L, Crujeiras AB, Irles E, Segues N, Bujanda L, Portillo MP. Comparative Effects of Pterostilbene and Its Parent Compound Resveratrol on Oxidative Stress and Inflammation in Steatohepatitis Induced by High-Fat High-Fructose Feeding. Antioxidants (Basel). 2020 Oct 24;9(11):1042. doi: 10.3390/antiox9111042. PMID: 33114299; PMCID: PMC7690896.
50. Hirsova P, Ibrahim SH, Bronk SF, Yagita H, Gores GJ. Vismodegib suppresses TRAIL-mediated liver injury in a mouse model of nonalcoholic steatohepatitis. PLoS One. 2013 Jul 22;8(7):e70599. doi: 10.1371/journal.pone.0070599. PMID: 23894677; PMCID: PMC3718793.
51. Verdelho Machado M, Diehl AM. Role of Hedgehog Signaling Pathway in NASH. Int J Mol Sci. 2016 Jun 1;17(6):857. doi: 10.3390/ijms17060857. PMID: 27258259; PMCID: PMC4926391.
52. Chaudhary NI, Roth GJ, Hilberg F, Müller-Quernheim J, Prasse A, Zissel G, Schnapp A, Park JE. Inhibition of PDGF, VEGF and FGF signalling attenuates fibrosis. Eur Respir J. 2007 May;29(5):976-85. doi: 10.1183/09031936.00152106. Epub 2007 Feb 14. PMID: 17301095.
53. Nakamura I, Zakharia K, Banini BA, Mikhail DS, Kim TH, Yang JD, Moser CD, Shaleh HM, Thornburgh SR, Walters I, Roberts LR. Brivanib attenuates hepatic fibrosis in vivo and stellate cell activation in vitro by inhibition of FGF, VEGF and

PDGF signaling. PLoS One. 2014 Apr 7;9(4):e92273. doi: 10.1371/journal.pone.0092273. Erratum in: PLoS One. 2015;10(11):e0142355. PMID: 24710173; PMCID: PMC3977817.

54. Echeverría F, Valenzuela R, Espinosa A, Bustamante A, Álvarez D, Gonzalez-Mañan D, Ortiz M, Soto-Alarcon SA, Videla LA. Reduction of high-fat diet-induced liver proinflammatory state by eicosapentaenoic acid plus hydroxytyrosol supplementation: involvement of resolvins RvE1/2 and RvD1/2. J Nutr Biochem. 2019 Jan;63:35-43. doi: 10.1016/j.jnutbio.2018.09.012. Epub 2018 Sep 21. PMID: 30321750.

55. Ortiz M, Soto-Alarcón SA, Orellana P, Espinosa A, Campos C, López-Arana S, Rincón MA, Illesca P, Valenzuela R, Videla LA. Suppression of high-fat diet-induced obesity-associated liver mitochondrial dysfunction by docosahexaenoic acid and hydroxytyrosol co-administration. Dig Liver Dis. 2020 Aug;52(8):895-904. doi: 10.1016/j.dld.2020.04.019. Epub 2020 Jun 30. PMID: 32620521.

56. Lieber CS, Leo MA, Mak KM, Xu Y, Cao Q, Ren C, Ponomarenko A, DeCarli LM. Acarbose attenuates experimental non-alcoholic steatohepatitis. Biochem Biophys Res Commun. 2004 Mar 12;315(3):699-703. doi: 10.1016/j.bbrc.2004.01.116. PMID: 14975757.

57. Okada K, Yanagawa T, Warabi E, Yamastu K, Uwayama J, Takeda K, Utsunomiya H, Yoshida H, Shoda J, Ishii T. The alpha-glucosidase inhibitor acarbose prevents obesity and simple steatosis in sequestosome 1/A170/p62 deficient mice. Hepatol Res. 2009 May;39(5):490-500. doi: 10.1111/j.1872-034X.2008.00478.x. Epub 2009 Jan 16. PMID: 19207582.

58. Yamagishi S, Nakamura K, Inoue H. Acarbose is a promising therapeutic strategy for the treatment of patients with nonalcoholic steatohepatitis (NASH). Med Hypotheses. 2005;65(2):377-9. doi: 10.1016/j.mehy.2005.01.032. PMID: 15922116.

59. Amorim R, Cagide F, Tavares LC, Simões RF, Soares P, Benfeito S, Baldeiras I, Jones JG, Borges F, Oliveira PJ, Teixeira J. Mitochondriotropic antioxidant based on caffeic acid AntiOxCIN4 activates Nrf2-dependent antioxidant defenses and quality control mechanisms to antagonize oxidative stress-induced cell damage. Free Radic Biol Med. 2022 Feb 1;179:119-132. doi: 10.1016/j.freeradbiomed.2021.12.304. Epub 2021 Dec 22. PMID: 34954022.

60. Ziamajidi N, Khaghani S, Hassanzadeh G, Vardasbi S, Ahmadian S, Nowrouzi A, Ghaffari SM, Abdirad A. Amelioration by chicory seed extract of diabetes- and oleic acid-induced non-alcoholic fatty liver disease (NAFLD)/non-alcoholic steatohepatitis (NASH) via modulation of PPAR α and SREBP-1. Food Chem Toxicol. 2013 Aug;58:198-209. doi: 10.1016/j.fct.2013.04.018. Epub 2013 Apr 18. PMID: 23603006.

61. Zhong XC, Liu YM, Gao XX, Krausz KW, Niu B, Gonzalez FJ, Xie C. Caffeic acid

phenethyl ester suppresses intestinal FXR signaling and ameliorates nonalcoholic fatty liver disease by inhibiting bacterial bile salt hydrolase activity. Acta Pharmacol Sin. 2022 Jun 2. doi: 10.1038/s41401-022-00921-7. Epub ahead of print. PMID: 35655096.

62. Rangwala F, Guy CD, Lu J, Suzuki A, Burchette JL, Abdelmalek MF, Chen W, Diehl AM. Increased production of sonic hedgehog by ballooned hepatocytes. J Pathol. 2011 Jul;224(3):401-10. doi: 10.1002/path.2888. Epub 2011 May 5. PMID: 21547909; PMCID: PMC3628812.

63. Verdelho Machado M, Diehl AM. Role of Hedgehog Signaling Pathway in NASH. Int J Mol Sci. 2016 Jun 1;17(6):857. doi: 10.3390/ijms17060857. PMID: 27258259; PMCID: PMC4926391.

64. Syn WK, Choi SS, Liaskou E, Karaca GF, Agboola KM, Oo YH, Mi Z, Pereira TA, Zdanowicz M, Malladi P, Chen Y, Moylan C, Jung Y, Bhattacharya SD, Teaberry V, Omenetti A, Abdelmalek MF, Guy CD, Adams DH, Kuo PC, Michelotti GA, Whitington PF, Diehl AM. Osteopontin is induced by hedgehog pathway activation and promotes fibrosis progression in nonalcoholic steatohepatitis. Hepatology. 2011 Jan;53(1):106-15. doi: 10.1002/hep.23998. Epub 2010 Oct 21. PMID: 20967826; PMCID: PMC3025083.

65. Mauer AS, Hirsova P, Maiers JL, Shah VH, Malhi H. Inhibition of sphingosine 1-phosphate signaling ameliorates murine nonalcoholic steatohepatitis. Am J Physiol Gastrointest Liver Physiol. 2017 Mar 1;312(3):G300-G313. doi: 10.1152/ajpgi.00222.2016. Epub 2016 Dec 30. PMID: 28039158; PMCID: PMC5401989.

66. Yoshida T, Tsuchiya A, Kumagai M, Takeuchi S, Nojiri S, Watanabe T, Ogawa M, Itoh M, Takamura M, Suganami T, Ogawa Y, Terai S. Blocking sphingosine 1-phosphate receptor 2 accelerates hepatocellular carcinoma progression in a mouse model of NASH. Biochem Biophys Res Commun. 2020 Oct 1;530(4):665-672. doi: 10.1016/j.bbrc.2020.07.099. Epub 2020 Aug 4. PMID: 32768187.

67. Liao CY, Song MJ, Gao Y, Mauer AS, Revzin A, Malhi H. Hepatocyte-Derived Lipotoxic Extracellular Vesicle Sphingosine 1-Phosphate Induces Macrophage Chemotaxis. Front Immunol. 2018 Dec 19;9:2980. doi: 10.3389/fimmu.2018.02980. PMID: 30619336; PMCID: PMC6305739.

68. Duan J, Wang Z, Duan R, Yang C, Zhao R, Feng Q, Qin Y, Jiang J, Gu S, Lv K, Zhang L, He B, Birnbaumer L, Yang S, Chen Z, Yang Y. Therapeutic targeting of hepatic ACSL4 ameliorates NASH in mice. Hepatology. 2022 Jan;75(1):140-153. doi: 10.1002/hep.32148. Epub 2021 Nov 27. PMID: 34510514; PMCID: PMC8688219.
